# EGFR: New Insights on Its Activation and Mutation in Tumor and Tumor Immunotherapy

**DOI:** 10.1002/advs.202505785

**Published:** 2025-08-27

**Authors:** Yuanzhuo Gu, Hongchao He, Shilei Qiao, Yifan Shao, Lurong Wang, Zhengkui Zhang, Long Zhang, Fangfang Zhou

**Affiliations:** ^1^ Department of Gynecological Oncology Women's Hospital Zhejiang University School of Medicine Hangzhou 310006 China; ^2^ The First Affiliated Hospital the Institutes of Biology and Medical Sciences Suzhou Medical College Soochow University Suzhou Jiangsu 215006 China; ^3^ Life Sciences Institute and State Key Laboratory of Transvascular Implantation Devices of the Second Affiliated Hospital of Zhejiang University School of Medicine Zhejiang University Hangzhou 310009 China; ^4^ The MOE Basic Research and Innovation Center for the Targeted Therapeutics of Solid Tumors The First Affiliated Hospital Jiangxi Medical College Nanchang University Nanchang 330006 China; ^5^ Frontiers Medical Center Tianfu Jincheng Laboratory Chengdu 610093 China

**Keywords:** cancer immunotherapy, combination therapy, epidermal growth factor receptor (EGFR), tumor microenvironment

## Abstract

The success of immune checkpoint blockades (ICBs) has accelerated the clinical implementation of multiple single agents and combination immunotherapies, but the response rates vary. Reconsideration of immune‐oncology therapeutic failures via perspective from tumor‐intrinsic (such as oncogenic driver genes) and tumor‐extrinsic (the complexity of immune cell–cancer cell interactions) may help to better design more effective anticancer drugs and treatment strategies. Herein, in this review, introducing the frequently mutated gene *EGFR* in tumors and its abnormal activation are mainly focused on, highlighting that epidermal growth factor receptor (EGFR) wild‐type and mutants respond differently to ICBs via tumor‐intrinsic and tumor‐extrinsic manners. Through briefly reviewing how EGFR is activated and the current EGFR targeting strategy, the present clinical trials of combination with EGFR inhibitors and ICBs are summarized, and the mechanism by which EGFR affects immunotherapy and measures to improve the efficacy of immunotherapy are discussed.

## Graphical abstract

This review provides new perspectives on EGFR's activation, mutations, and their impact on tumor immunotherapy. It summarizes how EGFR is activated and the current EGFR targeting strategy, evaluates their clinical outcomes in combination with immunotherapy, highlighting differential responses in tumors with EGFR wild‐type and different mutants. It delineates tumor‐intrinsic and extrinsic mechanisms and summarizes key insights into potential targeting strategies based on current evidence.

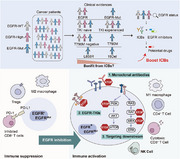

## 1 Introduction

Innovations in novel cancer immunotherapies, such as ICBs have greatly improved survival outcomes in the clinic.^[^ ^1^, ^2^ ^]^ However, tumors harboring different types of mutations respond differently to immunotherapy. In tumors with *KRAS* mutations (especially those with *TP53* mutations) and *BRAF* non‐V600E mutations, the use of ICBs can provide clinical benefits; however, tumor mutations such as *EGFR*, *ALK*, and *MET* are usually unresponsive to ICBs,^[^ ^3^ ^]^ showing resistance and escape.

EGFR belongs to the ErbB family of receptor tyrosine kinase (RTK), and is the most frequently mutated gene in human cancers. Activation and mutated EGFR are understood as a driving event in many types of tumors. Although multiple clinical trials revealed that patients with EGFR‐sensitive mutations gain better survival benefits from EGFR‐tyrosine kinase inhibitors (TKIs) than chemotherapy, the resistance to EGFR TKIs is inevitable and challenging, looking forward to innovative therapies. And compared with EGFR wild‐type (EGFR^wt^) tumors, EGFR mutant (EGFR^mut^) tumors are more heterogeneous, characterized tumor microenvironment (TME) and multiple tumor‐intrinsic factors. Whether ICBs are suitable for cancer patients with EGFR mutations remains to be explored. What is more, initial EGFR TKIs could induce antitumor immunity in different ways,^[^ ^4^, ^5^ ^]^ and it is worth revisiting the efficiency of their combination with ICBs.

In this review, we briefly reviewed the structural and functional organization of EGFR, how it is activated in a dependent or independent manner of ligand, different kinds of mutations, and the history of four generations of EGFR TKIs. And we mainly focused on summarizing the clinical data with regard to the efficacy of ICBs (either monotherapy or combination with other therapy, including chemotherapy, TKIs, and so on) in EGFR^wt^ and EGFR^mut^ patients, introducing how EGFR modulates the immune landscape to create niches for immune escape in both tumor‐intrinsic and extrinsic manners, and putting forward further combined strategies to improve the efficacy of ICBs in patients with abnormal EGFR signaling.

## 2 EGFR Activation and Mutations

### 2.1 The Structure and Functional Organization of EGFR

**Figure 1:**
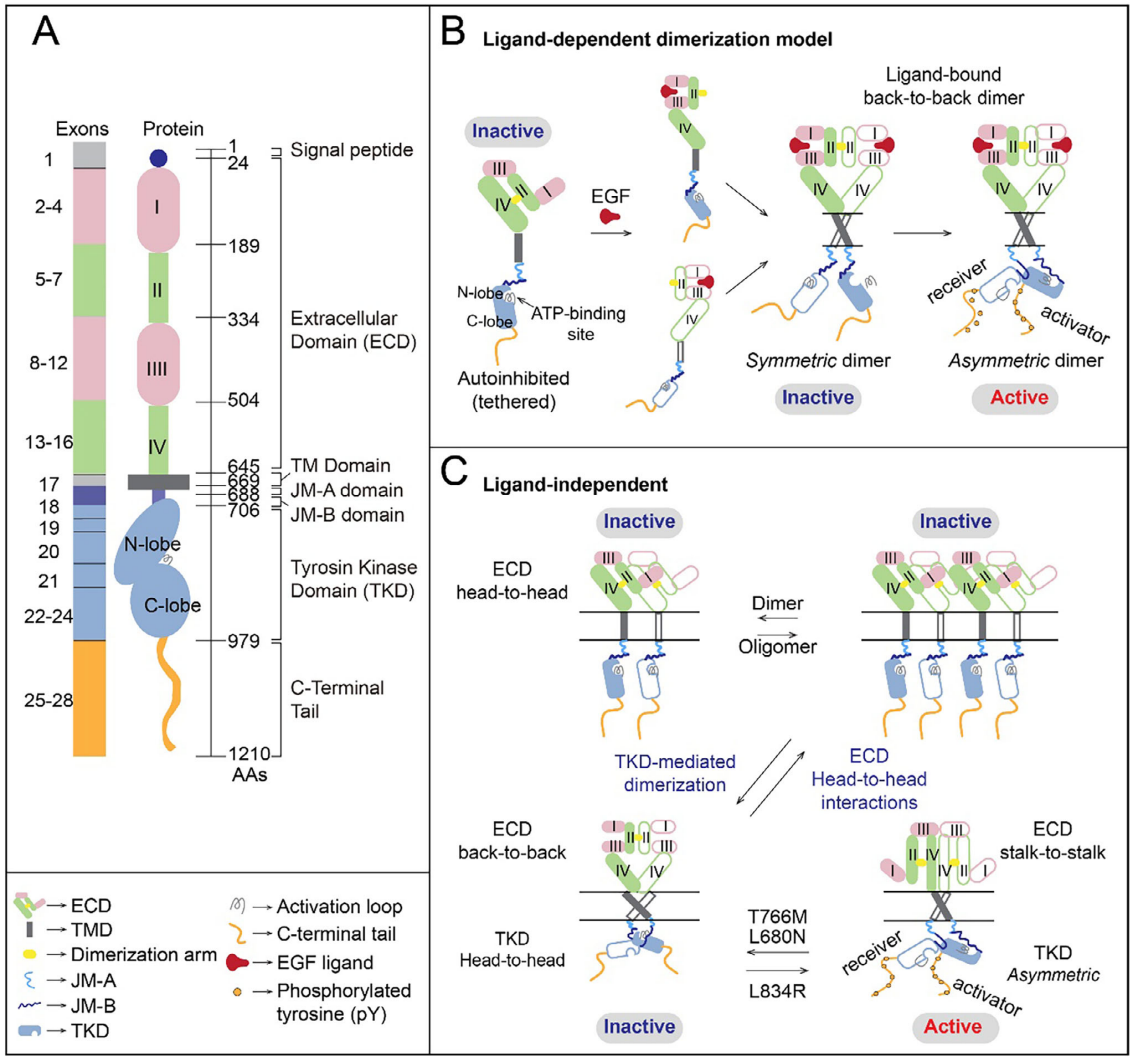
Domain architecture of EGFR and models of ligand‐free and ligand‐bound EGFR complexes. A) The extracellular domain (ECD) comprises 4 domains: I–IV, sometimes referred to as L1, cystine‐rich (CR)1, L2, and CR2. Domains I and III (pick) share ≈37% sequence identity, while domains II and IV (green) are cystine‐rich. The ECD is followed by a single membrane‐spanning region, a cytoplasmic tyrosine kinase domain (TKD), and a variable‐length tail (orange) that harbors several phosphorylation sites. The N‐lobe and C‐lobe of the TKD are in blue. This color scheme is used in all figures unless otherwise noted. Amino acid numbers are noted for each domain boundary. B) Models of ligand‐dependent EGFR dimerization. The monomeric EGFR (left) displays the structure of the receptor in the absence of ligand. The ECD adopts a tethered conformation in which the dimerization arm is buried inside the protein, forming an intramolecular bridge between domains II and IV. Upon ligand binding to domains I and III, the ECD undergoes a large rearrangement, resulting in the formation of the ligand‐binding pocket and exposure of the dimerization arm that is now ready to form an intermolecular bridge with another EGFR whose extracellular domain also adopts this extended conformation. Compared with the inactive status of the symmetric dimer in TKDs, in the active receptor dimer, the TM domain form a dimer stabilized by the N‐terminal dimerization motif, the JM domain dissociates from anionic phospholipids of the membrane, and the TKDs form an asymmetric dimer. C) Models of ligand‐independent EGFR structure. 1) Top: Autoinhibited ligand‐free receptors form dimers (left) and larger oligomers (right) via extracellular head‐to‐head interactions, and TKDs remain as non‐interacting units; 2) Bottom: A ligand‐free back‐to‐back ECD subunit coupled to a head‐to‐head TKD subunit (left), and a ligand‐free stalk‐to‐stalk ECD subunit coupled to the asymmetric TKD subunit (right). Kinase‐mediated receptor dimerization outcompetes head‐to‐head interactions to form two types of receptor dimers that typically coexist in equilibrium (bearing asymmetric TKD and symmetric TKD dimer configurations). Head‐to‐head dimers and oligomers are disrupted by kinase‐mediated dimerization independently of whether the driver mutation. The L680N or T766M kinase domain mutation shifts the equilibrium toward the dimer population bearing symmetric TKD dimers while L834R shifts the equilibrium toward the dimer population bearing the asymmetric TKD dimer.

The EGFR family of RTKs comprises four members: EGFR itself, ErbB2 (HER2/Neu), ErbB3 (HER3), and ErbB4 (HER4). Human *EGFR*, encoded by 28 exons on chromosome 7, consists of 1210 amino acids with an approximate molecular mass of 134 kDa.^[^ ^6^ ^]^ The initial 24 amino acids form the signal peptide of the EGFR protein, commonly excluded from the structural numbering, but included when defining EGFR cancer mutations. EGFR is a single‐pass transmembrane protein, composed of a large extracellular domain (ECD), a transmembrane (TM) domain, an intracellular juxtamembrane (JM) domain, a tyrosine kinase domain (TKD), and a C‐terminal regulatory tail (**Figure** **1**A).

The ECD (residues 25‐645) contains two homologous ligand‐binding domains (I and III, also referred to as L1 and L2) with a β‐helix solenoid structure, along with two cystine‐rich domains (II and IV, also called CR1 and CR2) held together by disulfide bonds.^[^ ^7^ ^]^ The TM domain (646‐668) serves as a bridge connecting the extracellular and intracellular domains of EGFR. Studies have reported that the pivoting and rotational motion of the TM helices determined by the thickness of the bilayer^[^ ^8^, ^9^ ^]^ affects the allosteric modulation of EGFR.^[^ ^9^, ^10^ ^]^ The JM domain (669‐705) links the C‐terminus of the TM domain to the kinase domain of EGFR and functions in the dimerization and activation of EGFR.^[^ ^11^, ^12^, ^13^ ^]^ Specifically, the N‐terminal of JM (residues 669‐687, referred to as JM‐A) forms an antiparallel dimer through a conserved LRRLL sequence, while the C‐terminal of JM (residues 688‐705, referred to as JM‐B) interacts with the kinase domains.^[^ ^11^, ^14^, ^15^ ^]^

The TKD (706‐978) can be further divided into two parts: the NH2‐terminal lobe (N‐lobe) and the larger COOH‐terminal lobe (C‐lobe). The N‐lobe consists of five β‐sheet strands and 1 α C‐helix spanning from residue 729‐744, while the C‐lobe is composed of five α helices. The ATP‐binding site is located in a cleft between N‐ and C‐lobe, beneath a highly conserved glycine‐rich phosphate‐binding loop.^[^ ^16^ ^]^

The final part is the C‐terminal domain (982‐1210), which regulates EGFR activation by suppressing kinase activity in the absence of autophosphorylation.^[^ ^17^, ^18^ ^]^ This domain contains numerous proline residues for phosphorylation sites^[^ ^19^ ^]^ and commonly displays disordered regions in the published crystal structures of EGFR.^[^ ^20^ ^]^ Upon ligand binding, the C‐terminal tail undergoes tyrosine phosphorylation, facilitating interactions between the receptor and downstream effectors such as Src homology 2 domain‐containing adaptor protein 1 (Shc1) and growth factor receptor‐bound protein 2 (Grb2).^[^ ^21^ ^]^

### 2.2 EGFR Activation by Ligand‐Bound

The conventional understanding of EGFR activation posits that the monomeric receptor converts to a dimeric form upon ligand binding, typically it concludes three sequential steps: 1) Ligands binding, specific ligands such as EGF, transforming growth factor α (TGFα), amphiregulin (AR), βcellulin (BTC), epigen (EPN), epiregulin (EPR) and heparin‐binding EGF‐like growth factor (HB‐EGF) bind to the extracellular domain of EGFR,^[^ ^22^ ^]^ which prompts the formation of both homodimers and heterodimers; 2) Dimerization, in the dimerized conformation, the EGFR TKD can be phosphorylated in the presence of adenosine triphosphate (ATP); 3) Phosphorylation, it triggers the auto‐phosphorylation of specific tyrosine kinase residues within the cytoplasmic regulatory domain, thereby activating downstream signaling pathways,^[^ ^23^ ^]^ including RAS‐RAF‐MEK‐ERK, phsphoinositide‐3‐kinase (PI3K)/Akt serine/threonine kinase (AKT) and Janus kinase (JAK)/signal transducers and activators of transcription (STAT) pathways.

#### 2.2.1 Ligand‐Mediated Extracellular Domain Dimerization

Domains I and III of EGFR constitute the ligand‐binding site,^[^ ^24^, ^25^, ^26^ ^]^ while domain II contains a “dimerization arm” (colored yellow) that interacts with the corresponding element of the other subunit in a dimer and bridges the ligand‐binding domain.^[^ ^27^ ^]^ In the absence of ligand, the ECD adopts a tethered and auto‐inhibited conformation, with the “dimerization arm” buried. Upon ligand binding, Domain I, II and III form a C shape that refers as the head of the ECD, while domain IV forms an elongated leg, converting the receptor to a straightened form and releasing the dimerization arm to interact with another monomer B. Consequently, a back‐to‐back dimer is formed^[^ ^28^ ^]^ (Figure 1B left). Interestingly, different ligand activates EGFR with distinct signaling outcomes. For example, although both EGF and TGFα stabilize similar ECD dimers of EGFR, the conformation of TMD dimers and the arrangement of the JM domains differ significantly,^[^ ^29^, ^30^ ^]^ which leads to different dimer stability and longevity^[^ ^31^ ^]^ and, in turn, functions distinct tyrosine phosphorylation patterns in the C‐terminus.^[^ ^32^ ^]^

#### 2.2.2 Asymmetric Dimerization of Kinase Domains

In the intracellular kinase domains, EGFR activation involves an asymmetric dimer interaction in which one kinase serves as a cyclin‐like allosteric activator of the other.^[^ ^11^, ^15^, ^23^, ^33^ ^]^ Specifically, the C‐lobe of one kinase (the activator) contacts the N‐lobe of another kinase (the receiver), stabilizing the receiver in an active conformation^[^ ^23^ ^]^ (Figure 1B right).

There is a controversy regarding the cooperativity of the conformational changes of the TKD and the ECD, as well as their dimerization and ligand binding to monomeric and dimeric receptor species. One view suggests positive and cooperative ligand binding to EGFR.^[^ ^34^, ^35^ ^]^ According to this view, the inactive and active conformations of the TKD are respectively coupled to the tethered and untethered structures of the ECD, and different cooperativities are assumed,^[^ ^36^ ^]^ supported by a comprehensive and quantitative model.^[^ ^37^ ^]^ Conversely, another view proposes negative and cooperative ligand binding to EGFR, which is supported by negative cooperative EGF binding curves^[^ ^38^, ^39^ ^]^ and the observation of a squeezed, restrained ligand‐binding site in the unliganded receptor within a single liganded dimer in the Drosophila EGFR.^[^ ^40^ ^]^

### 2.3 EGFR Activation via Ligand‐Free

Recently, evidence has accumulated for ligand‐free EGFR dimers and oligomers over the years,^[^ ^35^, ^41^, ^42^, ^43^, ^44^, ^45^, ^46^, ^47^, ^48^ ^]^ but the mechanisms by which ligand‐independent activation occurs are still unclear.

Inactive EGFR can exist as monomers, dimers, and even polymers, but the domain formation of ECD and TKD varies depending on the context. Without ligands, EGFR can also form a head‐to‐head dimer or back‐to‐back dimer of ECD, and even multiple ligand‐free receptor polymer chains of various lengths assemble^[^ ^49^ ^]^ (Figure 1C top). In the intracellular domain, the architecture of the head‐to‐head interaction of TKD prevents the formation of active, asymmetric TKD dimers,^[^ ^50^ ^]^ which leads to the inactive EGFR. Only cells with some mutations or treated with type I TKIs will split the autoinhibited head‐to‐head polymers of TKD to form a stalk‐to‐stalk flexible non‐extended dimers^[^ ^51^ ^]^ (Figure 1C bottom), which activate asymmetric tyrosine kinase dimers, supported by the electron microscopy (EM) images of purified, near‐full‐length Δ998‐EGFR.^[^ ^51^, ^52^ ^]^ Alternatively, molecular dynamics (MD) simulations^[^ ^53^ ^]^ suggested that the symmetric TKD dimer is coupled via a C‐crossing TM domain dimer to a ligand‐free back‐to‐back dimer,^[^ ^25^ ^]^ analogous to the X‐ray structure of the Drosophila ECM dimer^[^ ^40^ ^]^ and a model based on small‐angle X‐ray scattering (SAXS) data from Caenorhabditis elegans EGFR.^[^ ^44^ ^]^

Except for the EGFR activation via dimerization in a ligand‐free manner, it can be also activated via multimerization. A model of EGFR multimerization through self‐association of ligand‐bound dimers showed that the majority of kinase domains are activated cooperatively, thereby boosting tail phosphorylation.^[^ ^54^ ^]^

### 2.4 EGFR Activation via Overexpression and Mutations in Cancers

Mutations or deletions in EGFR also result in ligand‐independent receptor activation in certain cancers.^[^ ^55^, ^56^, ^57^ ^]^ Especially drug‐resistant EGFR mutations also facilitate the formation of ligand‐independent, kinase‐active oligomers, which in turn promote and stabilize the assembly of oligomer‐dependent active dimer subunits, thereby bypassing the requirement for ligand binding. Among them, active dimer sub‐units within ligand‐free oligomers are the high‐affinity binding sites to promote tumor growth.^[^ ^58^ ^]^ Several mechanisms can disrupt the stringent control of EGFR signaling, such as increased ligand production, overproduction of EGFR protein, mutations that result in the continuous activation of EGFR, impaired downregulation of EGFR, and the activation of EGFR via interactions with other cell‐surface receptors.^[^ ^59^ ^]^ In this review, we mainly focused on the overproduction of EGFR and mutations causing constitutive activation of EGFR.

#### 2.4.1 EGFR Overexpression

EGFR overexpression is the result of 1) Amplification of the *EGFR* gene, observed in various cancers including breast cancer,^[^ ^60^ ^]^ non‐small‐cell lung cancer (NSCLC),^[^ ^61^ ^]^ and glioblastoma multiforme;^[^ ^62^, ^63^ ^]^ 2) Overproduction at the transcriptional level. For example, *EGFR* transcription was activated by wild‐type and mutant p53 proteins binding to its promoter directly^[^ ^64^, ^65^ ^]^ or with a specific intronic enhancer region.^[^ ^66^, ^67^ ^]^ Additionally, the presence of variable CA dinucleotide repeats within intron 1 of the *EGFR* gene,^[^ ^68^ ^]^ near the enhancer region, appears to play a role in modulating EGFR transcription, with a decrease in transcription observed as the number of CA repeats increases;^[^ ^69^, ^70^ ^]^ 3) Enhanced recycling of the EGFR back to the cell surface following EGF stimulation, which was observed in the laryngeal papilloma cells with high levels of EGFR;^[^ ^71^ ^]^ 4) Enhanced expression of EGFR also drive dimerization through mass activation, which is important in certain cancers.^[^ ^72^ ^]^

#### 2.4.2 Mutations that Constitutively Activate EGFR

##### Mutations in the ECD

**Figure 2:**
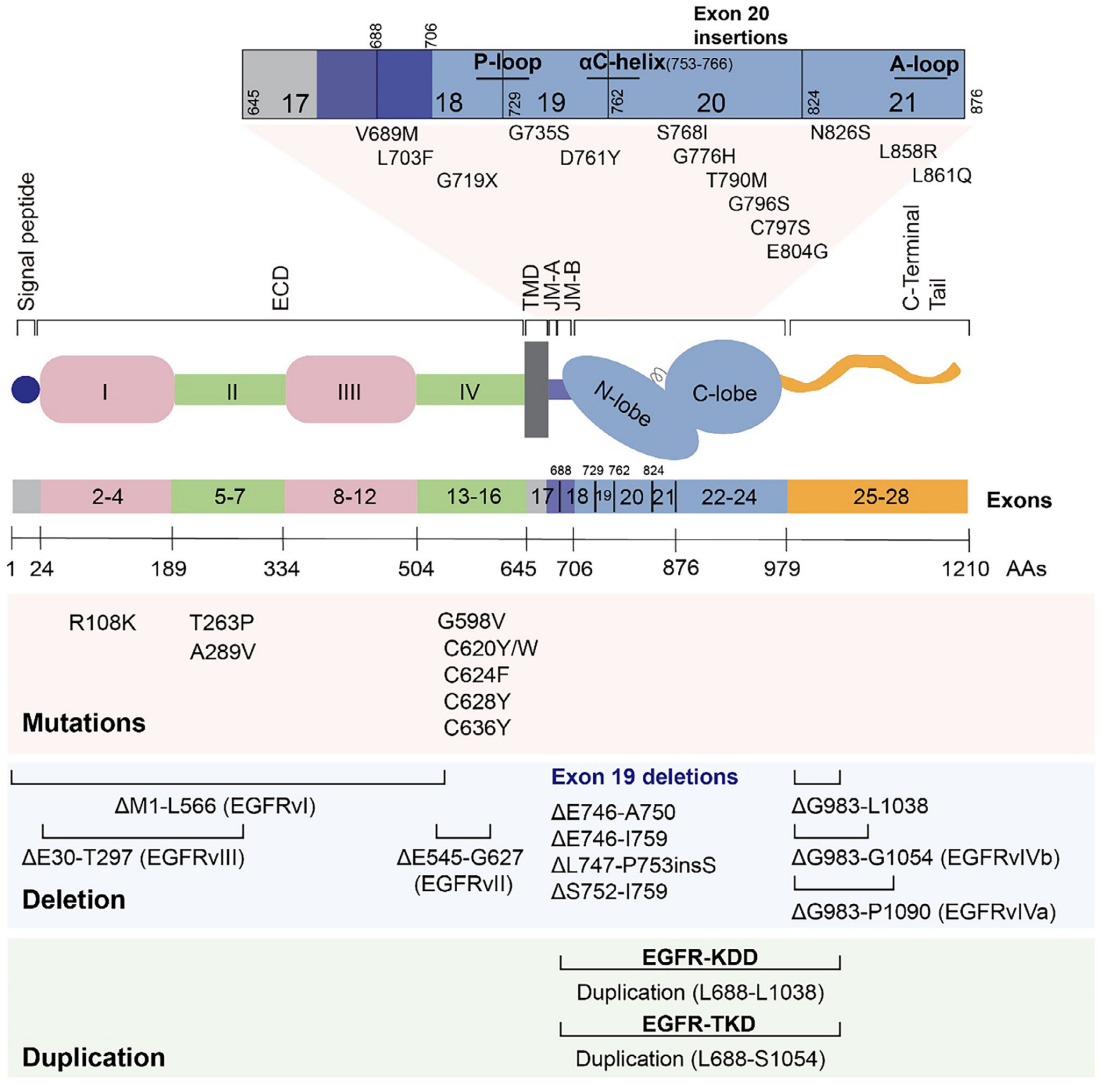
A subset of major EGFR mutations derived from cancer patients is presented according to the location of each exon. Schematic depicting the different exons in EGFR and the functional domains in which the sensitizing or resistance‐conferring mutations occur, including EGFR mutations, deletion, and duplication.

Abundant EGFR mutants have been identified in different cancers, summarized in **Figure** **2**. It is frequently observed that deletions of EGFR ECD occur in glioblastoma (GBM), including EGFRvI, vII, and vIII. EGFRvI lacks most of ECD from residue 1 to 566 and is tumorigenic.^[^ ^73^ ^]^ EGFRvIII has an in‐frame deletion of exons 2–7 in Domain I and II from residue 30 to 297 and is constitutively phosphorylated without ligand binding,^[^ ^74^ ^]^ which activates the c‐Jun N‐terminal kinase via PI3K,^[^ ^75^ ^]^ but cannot activate STAT1 and 3.^[^ ^76^, ^77^ ^]^ Due to the failure of ubiquitination of EGFRvIII, the oncogenic signaling was prolonged.^[^ ^78^ ^]^ EGFRvII has an in‐frame deletion of exons 14‐15 in Domain IV, residues 545 to 627, and is also constitutively phosphorylated without ligand binding.^[^ ^79^ ^]^ There is a hypothesis to explain how these mutants with deletions in the EGFR ECD are constitutively phosphorylated. 1) Lack of N‐terminal ECD leads to an unstable conformation of the intercellular domain, which promotes an asymmetric active TKD configuration with constitutive tyrosine phosphorylation at a limited level^[^ ^80^ ^]^; 2) Lack of ECD may induce rotation of the TMD, which rearranges the TKD dimer for partial activation.^[^ ^81^ ^]^

Mutations including R108K, T263P, A289V, and G598V^[^ ^82^, ^83^ ^]^ were found in GBM and glioblastoma cell lines and showed phosphorylated and responsiveness to the ligand. One possibility is that these mutations occur at the interface between domains I and II, or before the “β‐hairpin” that contacts domain IV in the inactive ECD conformation, which causes the conformation change by releasing the dimerization arm between domains II and IV, inducing ligand‐free phosphorylation of the EGFR mutants. Besides, mutations in domain IV, including C620Y/M, C624F, C628Y, and C636Y, were found through sequencing of EGFR in GBM patients^[^ ^81^, ^84^ ^]^ and displayed enhanced phosphorylation of EGFR via inducing disulfide bridge formation and, in turn, rotating the TMD.^[^ ^81^ ^]^

##### Mutations in the JM Domain

Mutations of V689M and L703F in the JM region were observed in NSCLC patients and constitutively activate mutant EGFR without ligand binding.^[^ ^11^ ^]^ The mechanism of the possible structural basis is V689 and L703 mutations can stabilize the asymmetric active dimeric form of TKD. The potential structural mechanism of EGFR activation by V689M mutation is that the side chain of a methionine residue substituted V689 extends into a cavity on the surface of the C‐lobe, and phenylalanine residue substituted L703 could aid to packing of the JM‐B of the “receiver” kinase with the C‐lobe of the “activator” kinase, which both may stabilize an asymmetric active conformation of TKD dimer.

##### Mutations in the TKD

Gly 719, located in the adjacent phosphate‐binding “P‐loop” of N‐lobe in the TKD, arches over the triphosphate moiety of the ATP substrate and participates in its coordination, which leads to the interactions holding helix αC in the inactive conformation.^[^ ^85^ ^]^ When it is substituted with serine, the inactive conformation of the P‐loop is destabilized, and therefore, the kinase is activated. And in NCSLC, G719 can also be substituted for alanine or cysteine.^[^ ^86^ ^]^ Deletion mutants in EGFR exon 19 (including E746 to I759) were observed in NSCLC patients,^[^ ^87^ ^]^ and Δ746‐750 deletion was a constitutively active mutant EGFR without ligand binding.^[^ ^88^ ^]^ When the murine hematopoietic Ba/F3 cell line expressed another 2 *EGFR* mutations, including Δ747‐753insS deletion^[^ ^57^, ^89^ ^]^ associated with the insertion of a serine residue and Δ752‐759 deletion,^[^ ^87^ ^]^ they all displayed enhanced tyrosine phosphorylation levels in a ligand‐independent manner.^[^ ^90^ ^]^ The potential structural mechanism of EGFR activation by deletion mutants from the N‐terminus of helix αC disrupted the autoinhibited TKD conformation via the release of the A‐loop from the active site for substrate binding.^[^ ^23^, ^90^ ^]^

Point mutation R776H was found in the lung cancer^[^ ^91^ ^]^ and constitutively autophosphorylated without ligand binding via transitions from an inactive αC‐helix “out” conformation to an active αC‐helix “in” conformation,^[^ ^92^ ^]^ which preferentially adopts the “receiver” position priming the N‐lobe interface for the dimerization, similar as the L858R and T790M mutations described below.

G735S, G796S, and E804G missense mutations identified in prostate cancer are oncogenic and constitutively activated in the absence of the ligand.^[^ ^93^ ^]^ Mutations G735S and G796S were also found in thyroid cancer and squamous cell carcinoma of the head and neck, respectively.^[^ ^94^, ^95^ ^]^ The G735 residue situated in the N‐lobe is thought to anticipated to induce a conformational shift, facilitating TKD activation. G796 and E804 are positioned at the interface of a symmetrically inactive dimer, potentially playing a role in the formation of such inactive dimers. The mutations G796S and E804G are speculated to undermine the stability of the inactive dimer, promoting a transition to an asymmetrically active conformation.

L858R and L861Q missense mutations in the A‐loop of TKD are also identified in the NSCLC.^[^ ^83^, ^96^ ^]^ The side chains of L858 and L861 play a role in creating a hydrophobic core, stabilized by residues from the helix αC, the A‐loop, and other regions.^[^ ^23^, ^97^ ^]^ Similar to R776H, L858R, and L861Q mutations shift the equilibrium of TKD into an active configuration by destabilizing the set of hydrophobic interactions.^[^ ^85^, ^90^ ^]^ Besides, a tandem kinase domain duplication of EGFR (named EGFR‐TKD) of resides 688‐1054 was found in GBM,^[^ ^82^, ^98^ ^]^ which showed enhanced basal autophosphorylation.^[^ ^99^ ^]^ Another kind of kinase domain duplication from residues 688 to 1038 (named EGFR‐KDD) was found in the lung, brain, and other cancers, which is oncogenic and constitutively activated by virtue of asymmetric intramolecular dimerization.^[^ ^100^ ^]^

##### Mutations in the C‐Terminal Tail Region

It is frequently observed that deletions of EGFR in glioblastoma, not only in the ECD, but also in the C‐terminal tail region, are collectively termed EGFRvIV. EGFRvIVa lacks exons 25‐27 (from residues G983 to P1090), and EGFRvIVb lacks exons 25 and 26 (from residues G983 to G1054).^[^ ^101^ ^]^ These two mutants displayed oncogenic and ligand‐independent constitutive activation,^[^ ^102^ ^]^ likely through a mechanism that relieves a restraining molecular fold, along with stabilization due to association with HSP90. Another deletion in exon 25 (from G983 to L1038) was identified in the GBM and NSCLC patients, which was also constitutively phosphorylated and caused STAT3 activation in the absence of the ligand.^[^ ^56^, ^103^ ^]^ Exon 25 spanning from G983 to L1038 encompasses the AP‐2 helix and the electrostatic hook, both of which contribute to the stabilization of the symmetric inactive TKD configuration.^[^ ^15^ ^]^ Consequently, the deletion of exon 25 is likely to disrupt the stability of the inactive TKD, thereby shifting the balance of the TKD toward an asymmetric active configuration within the dimeric structure.

In summary, EGFR overexpression and mutants appear to activate in diverse ways, which can occur independently of dimerization and also result in the activation of signaling networks in the absence of C‐terminal autophosphorylation. Among cancer patients, almost *EGFR* mutations are classical, either exon 19 deletions (Ex19Del) and/or L858R with or without T790M, while atypical EGFR mutations occur primarily in exons 18‐20, such as G719X and L861Q.^[^ ^104^ ^]^ The underlying molecular mechanisms and the functional implications of these non‐canonical characteristics of mutant EGFR warrant further investigation in future research.

## 3 Current Landscape of Targeting EGFR for Cancer Immunotherapy

### 3.1 Overview of Cancer Immunotherapy

Cancer immunotherapy refers to an exogenous intervention in the body's immune system, restarting and maintaining the “tumor immune” cycle, enhancing the body's antitumor immune response, strengthening the recognition and killing ability of tumor cells, and thus controlling or even specifically clearing tumors as a treatment method.

Currently, cancer immunotherapy concludes five categories,^[^ ^105^ ^]^ namely 1) Oncolytic viruses, a type of tumor killing virus with replication ability, which can target and reach tumor cells, replicate extensively inside the tumor cells and ultimately lyse the tumor cells, at the same time triggering an immune response to attract more immune cells to continue killing the remaining tumor cells^[^ ^106^ ^]^; 2) Cancer vaccines, the principle is that tumor antigens are introduced into the patient's body in various forms, such as tumor cells, tumor related proteins or peptides, genes expressing tumor antigens, etc., to overcome the immune suppression caused by tumors and enhance immunogenicity, patient's own immune system will be activated to induce cellular and humoral immune responses and achieve the goal of controlling or clearing tumors^[^ ^107^ ^]^; 3) Cytokine therapy, which cytokines are introduced into tumors to stimulate the body's immune system to kill tumor cells for tumor treatment; 4) Adoptive cell transfer therapy (ACT), which refers to the process of culturing and modifying collected patient's own immune cells in vitro to enhance their targeted killing function, and then reintroducing them back into the patient's body to eliminate tumor cells^[^ ^108^ ^]^; 5) ICBs therapy, whose main function is to block the interaction between tumor cells expressing immune checkpoints and immune cells, thereby blocking the inhibitory effect of tumor cells on immune cells.^[^ ^109^ ^]^ PD‐(L)1, cytotoxic T‐lymphocyte‐associated protein (CTLA‐4), T‐cell immunoglobulin and mucin‐domain containing‐3 (TIM3), and lymphocyte activation gene‐3 (LAG3) are the most studied currently.

### 3.2 Current EGFR Targeting Drugs and Targeting Mechanism

Broadly, there are two primary categories of EGFR family inhibitors: 1) Monoclonal antibodies that bind to the extracellular portion of the receptor,^[^ ^110^ ^]^ Cetuximab and Panitumumab are FDA‐approved antibodies used as part of standard of care in certain cancer treatment^[^ ^111^ ^]^; and 2) Small molecule inhibitors that target the intracellular tyrosine kinase domain,^[^ ^112^ ^]^ four generations of EGFR TKIs have been approved during last 2 decades. Despite the effectiveness of these agents, the major drawback of drug resistance has emerged. 3) Targeting the downstream signaling of EGFR is another promising novel therapeutic approach.

#### 3.2.1 Targeting EGFR at the Extracellular Domain

To date, 4 anti‐EGFR monoclonal antibodies named cetuximab, panitumumab, necitumumab, and nimotuzumab have been approved for clinical use,^[^ ^113^ ^]^ normally used in the head and neck squamous cell carcinoma (HNSCC), metastatic colorectal cancer (mCRC), and other solid tumors.

Cetuximab (IMC‐C225/ Erbitux) attaches to the domain III of EGFR, thereby inhibiting downstream signaling via the promotion of receptor internalization and obstruction of ligand–receptor binding. And the attachment of cetuximab to EGFR also generates a physical barrier that prevents EGFR from forming heterodimers with other members of the ErbB receptor family.^[^ ^114^ ^]^ Besides, as a human‐murine chimeric anti‐EGFR monoclonal antibody, cetuximab belongs to the IgG1 type, which can bind to Fc receptor on natural killer (NK) cells by its own Fc segment, thus producing antibody‐dependent cellular cytotoxicity (ADCC) effect.^[^ ^115^, ^116^ ^]^ Now, it was approved by the FDA in 2004 for the treatment of mCRC and approved in 2006 for treating locally or regionally advanced HNSCC.^[^ ^117^ ^]^ Normally, cetuximab is recommended for use in conjunction with different chemotherapy regimens, either as a first‐ or second‐line treatment option, or as a monotherapy for patients who have failed or are resistant to specific chemotherapy regimens. For example, compared with radiotherapy alone, cetuximab combined with radiation therapy improved overall survival (OS) in patients with locally or regionally advanced HNSCC (29.3 months vs 49.0 months).^[^ ^118^ ^]^ Similarly, cetuximab combined with platinum‐based chemotherapy with fluorouracil indeed prolonged OS and progression‐free survival (PFS) in recurrent locoregional or metastatic HNSCC.^[^ ^119^ ^]^

As a fully‐humanized monoclonal IgG2 antibody, panitumumab has a different binding epitope from cetuximab, which has nearly eight times stronger and more specific binding to EGFR.^[^ ^120^ ^]^ Panitumumab was first approved by the FDA in 2006 for EGFR‐expressing mCRC. Its monotherapy has been shown to extend PFS in patients with mCRC who have experienced disease progression during or after receiving chemotherapy regimens that include fluoropyrimidines, oxaliplatin, and irinotecan.^[^ ^121^, ^122^ ^]^ Similar to cetuximab, panitumumab treatment is also ineffective in mCRC harboring the mutated *RAS* gene.^[^ ^123^, ^124^ ^]^

Necitumumab has been approved for the first‐line treatment in patients with refractory metastatic squamous NSCLC, when it is combined with gemcitabine and cisplatin indeed to improve OS and PFS.^[^ ^125^, ^126^ ^]^ As a humanized monoclonal antibody, Nimotuzumab (h‐R3) has gained approval for the treatment of patients with HNSCC, glioma, and nasopharyngeal cancer in certain countries.^[^ ^127^, ^128^ ^]^ However, it has not been endorsed by the EMA and FDA for glioma treatment due to concerns over limited efficacy and a high incidence of adverse events.^[^ ^127^ ^]^

The four EGFR monoclonal antibody drugs were summarized in **Table** **1**, characterized by anti‐cancer features depending on their Fv and Fc regions. The main reasons underlying the resistance mechanism of EGFR monoclonal antibodies are as follows: 1) Genetic alterations in downstream signaling pathways, such as *RAS/RAF* and *PIK3CA* gene mutations. Activating mutations in *KRAS* or *NRAS* constitutively activate the mitogen‐activated protein kinase (MAPK) pathway, similarly driven by BRAF V600E, bypassing EGFR inhibition.^[^ ^129^, ^130^, ^131^, ^132^ ^]^ Activating mutations in *PIK3CA* sustain AKT signaling despite EGFR blockade.^[^ ^133^ ^]^ 2) EGFR structural modifications, EGFR extracellular domain mutations (e.g., S492R) preventing antibody binding,^[^ ^134^ ^]^ and cetuximab could bind to truncated EGFRvIII but did not modulate the proliferation or radiosensitivity of EGFR vIII‐expressing cancer cells.^[^ ^135^ ^]^ 3) Activation of compensatory RTKs. For instance, HER2/mesenchymal‐epithelial transition factor (MET) amplification^[^ ^136^ ^]^ and fibroblast growth factor receptor 1 (FGFR1), platelet‐derived growth factor receptor (PDGFR1), or vascular endothelial growth factor receptor 1 (VEGFR1) upregulation^[^ ^137^ ^]^ enable the bypassing of EGFR blockade. 4) Immunosuppressive niche remodeling, such as the accumulation of hyaluronic acid^[^ ^138^ ^]^ in the tumor microenvironment, can impede the entry of EGFR monoclonal antibodies and NK cells, leading to drug resistance.

**Table 1:** Therapeutic antibodies target EGFR in versatile ways.

Drugs	Antibody type	Mechanisms	Disease
Cetuximab (Erbitux, C225)	Chimeric IgG1 antibody	Competitively bind to the domain III (408–468) of EGFR with ligand, generate ADCC effect	HNSCC Metastatic CRC
Panitumumab (Vectibix, ABX‐EGF)	Humanized IgG2 antibody	Competitively bind to the domain III (386–391) of EGFR with ligand	Metastatic CRC Solid tumors
Necitumumab (portrazza)	Humanized IgG1 antibody	Competitively bind to the domain III (384–409) of EGFR with ligand	HNSCC Solid tumors
Nimotuzumab (h‐R3)	Humanized IgG1 antibody	Competitively bind to the domain III (353–358) of EGFR with ligand	HNSCC Metastatic pancreatic cancer Esophageal cancer Gastric cancer

Multiple clinical trials of 4 anti‐EGFR monoclonal antibodies have been performed in patients with different EGFR status (wild‐type or mutant) (**Table** **2**), but the results of different clinical trials are controversial.

**Table 2:** Clinical trials of the approved EGFR antibody drugs.

Tumor type	Phase	No. of EGFR (+)	Comparison (medication group and control group)	Efficacy (PR, OS, PFS et), survival benefit = months	Safety (list grade III and IV, adverse events)	Ref
Cetuximab						
mCRC	2	The efficacy of cetuximab has no obvious relationship with the level of EGFR in tumors. Perhaps the level of activated and phosphorylated EGFR or the mutation status of EGFR is more important for predicting efficacy	Cetuximab plus irinotecan (218) vs Cetuximab (111)	PR=22.9% (95% CI, 17.5–29.1) vs 10.8% (95% CI, 5.7–18.1)	Grade III and IV adverse events 65.1 vs 43.5%	[436]
CRC	3	Patients with a colorectal tumor bearing mutated K‐ras did not benefit from cetuximab, whereas patients with a tumor bearing wild‐type K‐ras did benefit from cetuximab	Cetuximab plus BSC (287) vs BSC (285)	OS = 6.1 vs 4.6 month OS (patients with WT K‐ras) = 9.5 vs 4.8 month PFS (patients with WT K‐ras) = 3.7 vs 1.9 month	Grade III and IV adverse events 78.5 vs 59.1%	[437] NCT00079066
Locoregionally advanced HNSCC	3	EGFR positive status was not the factor affecting the OS with the addition of cetuximab to radiotherapy, using forest plot	RT (213) vs RT plus Cetuximab (211)	OS= 29.3 vs 49 months	Received cetuximab and had a greater number of grade 3 and 4 infusion reactions (3%)	[118] NCT00004227
HNSCC	1	Panitumumab‐IRDye800CW and cetuximab‐IRDye800CW have toxicity and pharmacodynamic profiles that match the parent compound	Cetuximab‐IRDye800CW (12) vs Panitumab‐IRDye800CW (15)	–	Cetuximab‐IRDye800CW had 15 grade 1 AEs, and no grade 2 AEs	[438] NCT01987375 NCT02415881
Advanced NSCLC	3	EGFR FISH‐positivity was not predictive in the overall NSCLC patient population, but patients with squamous cell histology who were EGFR‐FISH+ benefit from cetuximab	Cetuximab vs control (with/without bevacizumab)	PFS in patients with EGFR‐FISH+ cancer=5.4 vs 4.8 month	Grade III and IV adverse events of decreased neutrophil count 37 vs 25%	[439] NCT00946712
Panitumumab						
Metastatic CRC	3	The favorable effect on PFS with panitumumab plus BSC was demonstrated irrespective of EGFR expression levels	Panitumumab plus BSC (231) vs BSC (232)	PR 8 vs 0%	Grade 3/4 skin toxicity (overall): 14 vs 0% Grade 3/4 hypomagnesemia: 3 vs 0%	[440]
Metastatic CRC	3	mCRC patients with WT KRAS benefit more from panitumumab plus FOLFOX4	Panitumumab plus FOLFOX4 vs FOLFOX4	PFS = 9.6 vs.8.0 months OS = 23.9 vs.19.7 months	Grade III and IV skin toxicity 96 vs 31%	[124]
KRAS wild‐type biliary cancer	2	Patients with WT KRAS did not benefit from panitumumab combined with chemotherapy, and the survival period of the combined treatment group was continuously lower than that of the control group.	Cisplatin + gemcitabine + Panitumumab vs Cisplatin + gemcitabine	PFS = 54 vs 73% OS = 12.8 vs 20.1 months	Neutropenia: 44% vs 47%	[441] NCT01320254
Confirmed metastatic colon or rectum adenocarcinoma	3	Patients with WT KRAS benefit from panitumumab plus BSC.	Panitumumab plus BSC (142) vs BSC (128)	PFS = 5.2 vs 1.7 months OS = 10.2 vs 7.4 months	Grade 3 and 4 adverse effects were 39.4% and 7.0% vs 15.6% and 3.1%	[442] NCT01412957
Nimotuzumab						
HNSCC	IIb	No correlation was found between EGFR expression and response at month 6 post‐treatment or survival at month 60 post‐treatment, adding nimotuzumab to traditional treatment can prolong the survival period of patients.	1. CRT + nimotuzumab (20) 2. CRT alone (20) 3. RT + nimotuzumab (17) 4. RT alone (19)	CR = 90 vs 70 vs 70.59 vs 31.01% PR = 10 vs 0 vs 5.88 vs 5.26% ORR = 100 vs 70 vs 76.47 vs 36.84%	Grade 3/4 mucositis: 55 vs 25 vs 59 vs 84%	[443]
Nasopharyngeal Carcinoma (NPC)	2	Total effective rate was more than 90% in locally advanced NPC by chemotherapy plus Nimotuzumab.	Chemotherapy vs sequential Nimotuzumab plus concurrent chemoradiotherapy	OS = 85.6% LRC = 97.8% PFS = 79.5% in chemotherapy plus Nimotuzumab.	Grade 3/4 Neutropenia (35.5) Thrombocytopenia (17.7) in chemotherapy plus Nimotuzumab.	[444]
Advanced esophageal squamous cell cancer (ESCC)	2	The TPN regimen is safe and effective in the treatment of advanced ESCC, improving ORR and OS. It is more effective than the nimotuzumab combined with 5‐FU and cisplatin regimen and can be used as a treatment option before surgery or radiotherapy for locally advanced ESCC.	Nimotuzumab combined with paclitaxel and cisplatin (TPN regimen)	RR = 51.8% DCR = 92.9% OS = 14.0 months	Alopecia (78.6%) Neutropenia (46.4%), nausea (48.3%)	[445] NCT01336049
Necitumumab						
NSCLC	2	Necitumumab with pac‐carb is effective in the first‐line treatment of advanced squamous NSCLC with controllable safety.	Necitumumab with Paclitaxel‐carboplatin (Pac‐carb) (110) vs Pac‐carb (57)	Median PFS = 5.4 vs 5.6 OS = 13.2 vs 11.2	Grade ≥3 AEs : 65.1% vs 69.1% Serious AEs: 39.6% vs 34.5%	[446] NCT01769391
NSCLC	3	Patients who have no progression after receiving necitumumab combined with gemcitabine–cisplatin chemotherapy can continue to receive necitumumab maintenance therapy to maintain survival benefits without causing an increase in unexpected AEs, which is also applicable to patients in the subgroup with EGFR‐expressing tumors.	Necitumumab Continuation (261) vs Gemcitabine–Cisplatin non‐progressors (215)	OS = 15.9 vs 15.0 months; PFS = 7.4 vs 6.9 months; OS (EGFR^+^) = 16.1 vs 14.9 months; PFS (EGFR^+^) = 7.4 vs 6.9 months	Grade ≥3 AEs : 70.9% vs 53% Serious AEs: 37.2% vs 22.8%	[447] NCT00981058

BSC, best supportive care; RT, radiotherapy; FOLFOX, folinic acid (leucovorin), fluorouracil, oxaliplatin; PR, Response rate; OS: Median overall survival; PFS, Median progression free survival; DCR, disease control rate; LCR, locoregional control; CRT, chemoradiotherapy; OR, Overall Response; IC, Investigator's choice

#### 3.2.2 Targeting EGFR at Intracellular Domain (4 Generations of EGFR TKIs)

##### First Generation of EGFR TKIs

**Figure 3:**
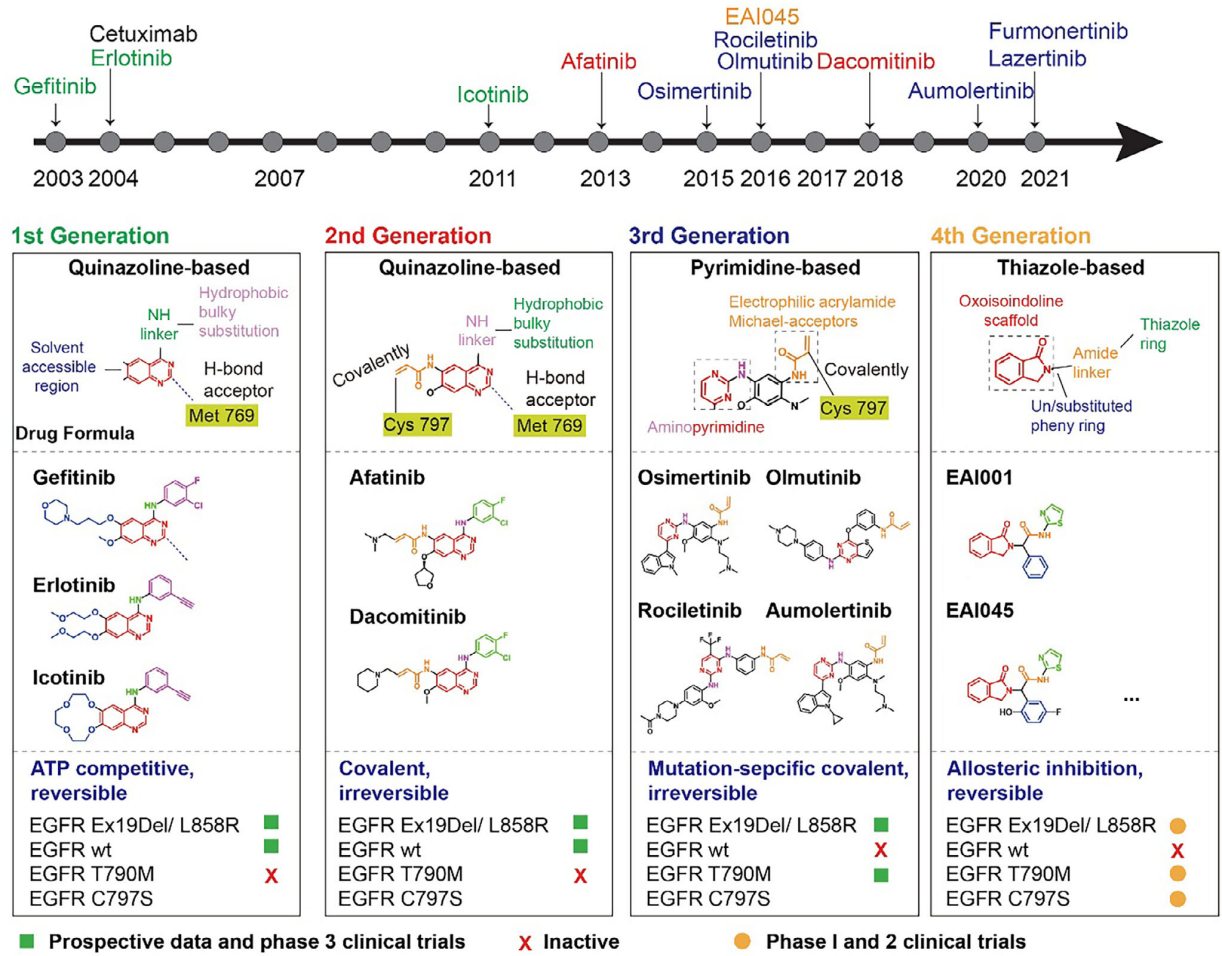
The development and comparative overview of EGFR inhibitors. Comparative overview of EGFR tyrosine‐kinase inhibitors (TKIs) and monoclonal antibodies in 2003–2021. Illustration of four generations of EGFR TKIs, highlighting their distinct molecular structures with drug formula, mechanisms of action, and activities of clinical trials with different EGFR status.

First‐generation of EGFR‐TKIs (e.g., gefitinib, erlotinib and icotinib) reversibly bind to EGFR and inhibit the binding of ATP to the tyrosine kinase domain, which shared standard structural features of four parts including i) solvent accessible region, ii) quinazoline central scaffold bound to the ATP binding site, iii) –NH–linker and iv) hydrophobic ring as bulky substitution responsible for the inhibition activity, all illustrated at **Figure** **3**.

Gefitinib (IressaTM, ZD‐1839) was the first EGFR TKIs approved by the FDA in 2003, which was used as the first line of treatment for lung cancer with mutated EGFR.^[^ ^139^, ^140^ ^]^ The N quinazoline ring of gefitinib forms a hydrogen bond with Met793 in the hinge region of EGFR. But due to the unfavorable phase III trial results, gefitinib was restricted in 2005 and withdrawn in 2012 by the FDA. In 2004, Erlotinib (TarcevaTM, CP‐358774) was approved by the FDA as the first‐line treatment of metastatic NSCLC with mutated EGFR, such as Ex19Del and L858R substitution.^[^ ^141^ ^]^ The mutation of the gatekeeper residue Thr790 leads to resistance against the first‐generation of EGFR TKIs, as it causes a steric hindrance with the aromatic ring of these drugs.^[^ ^142^ ^]^

Up to 50% patients treated with first‐generation EGFR‐TKIs develop acquired resistance through a *T790M EGFR* substitution mutation.^[^ ^143^, ^144^ ^]^

##### Second Generation of EGFR TKIs

In order to overcome the limitation of the first‐generation of EGFR‐TKIs, the second‐generation of EGFR‐TKIs (e.g., afatinib and dacomitinb) were developed, which have the same quinazoline scaffold as the first‐generation, but the side chain can irreversibly bind to Cys797 to inhibit the tyrosine kinase activity of EGFR. Moreover, these inhibitors establish two robust hydrogen bonds at the ATP binding site, one with Met769 and another with the mutated residue Met790, complemented by typical hydrophobic interactions with the hydrophobic pocket.^[^ ^145^ ^]^

Afatinib is the only one approved for the first‐line therapy for patients with metastatic NSCLC whose tumors contain Ex19Del or exon 21 (L858R) mutation to date.^[^ ^146^ ^]^ Unfortunately, most second‐generation inhibitors exhibited dose‐limiting toxicities, such as skin rash and gastrointestinal issues, along with an unacceptably low maximum tolerated dose (MTD), significantly limiting their therapeutic potential.^[^ ^147^, ^148^, ^149^ ^]^

##### Third Generation of EGFR TKIs

Third‐generation of EGFR‐TKIs (e.g., osimertinib and rociletinib) have represented potential effectiveness in NSCLC patients resistant to the first and second generations of EGFR TKIs,^[^ ^150^ ^]^ which is selective and irreversible, preferring to target mutant EGFR^T790M^ over wild‐type EGFR.^[^ ^151^ ^]^ Osimertinib (TagrissoTM, AZD‐9291) is the first TKI with a non‐quinazoline core approved by the FDA to be used as the adjuvant therapy for NSCLC patients with T790M mutation,^[^ ^152^ ^]^ but has less efficiency against wild‐type EGFR.^[^ ^151^, ^152^, ^153^ ^]^ Rociletinib (Xegafri^TM^, CO‐1686) is an irreversible inhibitor of mutated EGFR, which exhibited minimal efficacy against both wild‐type and exon‐20 insertion EGFR variants.^[^ ^154^, ^155^ ^]^

These inhibitors with an aminopyrimidine scaffold covalently bind to the active thiol group of Cys797 through their electrophilic acrylamide Michael acceptors. C797S mutation is considered the primary mechanism of resistance in all third‐generation EGFR TKIs,^[^ ^156^ ^]^ which precluded the formation of a covalent bond between the inhibitors and the cysteine residue at position 797 located on the edge of the ATP‐binding pocket.^[^ ^157^ ^]^ Besides, EGFRL718Q and EGFRL844V mutations also led to resistance to the third‐generation of EGFR TKIs.^[^ ^158^ ^]^

##### Fourth Generation of EGFR TKIs

In order to combat the C797S mutation, fourth‐generation of EGFR TKIs were required. EAI001 and EAI045 have therapeutic effects despite the C797S mutation, which were identified based on the screening of a large library of compounds targeting EGFR L858R/T790M.^[^ ^159^, ^160^ ^]^ EAI045 combined with cetuximab was related with significant tumor regression in L858R/T790M‐mutant mice,^[^ ^161^ ^]^ and also effective in L858R/T790M/C797S Ba/F3 cells and tumor xenografts. However, due to the peculiar structure at the allosteric pocket, EAI045 was ineffective against Ex19Del/T790M/C797S triple‐mutant cells.^[^ ^162^, ^163^ ^]^ In contrast, CH7233163^[^ ^164^ ^]^ was identified to overcome EGFR‐Ex19Del/T790M/C797S through multiple interactions with the αC helix conformation of EGFR, thereby achieving potent inhibitory activity and selectivity for the mutant.

Due to binding to the EGFR allosteric site and inducing conformational changes that decrease the affinity with ATP, EAIs display high selectivity.^[^ ^165^ ^]^ But the reduced efficacy of EAI045 in inhibiting EGFR dimers limited further development. Other allosteric inhibitors named JBJ‐04‐125‐02^[^ ^165^ ^]^ and JNJ‐09‐063^[^ ^166^ ^]^ were later developed, further supporting the combination therapy of EAI with ATP‐competitive TKIs.

Currently, there are a minimum of six fourth‐generation EGFR‐TKIs in early phase I clinical trials, including BLU‐701, BBT‐176, BLU‐945, TRX‐221, BPI‐361175, BDTX‐1535, characterized by their oral bioavailability, mutant selectivity, and thiazole amide structure.^[^ ^167^ ^]^ BBT‐176 was effective in patients with EGFR‐Ex19Del/T790M/C797S clinically, showing tumor shrinkage and radiological improvements.^[^ ^168^ ^]^

#### 3.2.3 Targeted Protein Degradation of EGFR Mutants

In order to overcome the EGFR‐TKIs resistance mediated by various EGFR mutants, EGFR‐targeted protein degradation (TPD) offers a novel strategy, which directs proteins to E3‐ubiqutin ligases, tagging them with ubiquitin chains for degradation. Currently, proteolysis targeting chimeras (PROTACs) and molecular glue (MG) degraders are two major modalities of TPD through ubiquitin–proteasome system.^[^ ^169^, ^170^ ^]^

EGFR‐targeting PROTACs consist of a ligand binding to the target protein EGFR and a ligand of an E3 ligase, recruiting an E3 ubiquitin ligase to degrade the EGFR mutant protein.^[^ ^171^, ^172^ ^]^ Due to the limited number of highly specific E3 ligands, only few E3 ligases have been developed for PROTACs, notably recruiting E3 ligases cereblon (CRBN), von Hippel‐Lindau (VHL), mouse double minute‐2 (MDM2), and inhibitor of apoptosis proteins (IAPs).^[^ ^173^ ^]^ In 2018, Crews et.al successfully developed the first VHL‐recruiting and gefitinib‐based EGFR‐PROTAC, which induced the degradation of EGFR Ex19Del in HCC‐827 cells and EGFR L858R in H3255 cells.^[^ ^171^ ^]^ Subsequently, PROTAC targeting EGFR double mutations such as L858R/T790M,^[^ ^174^, ^175^ ^]^ triple mutations (L858R/T790M/C797S) were developed,^[^ ^176^, ^177^ ^]^ respectively. Some of them showed good in vivo antitumor efficacy in preclinical models,^[^ ^178^ ^]^ including HJM‐561,^[^ ^179^ ^]^ compound 13 (dacomitinib‐based EGFR degraders),^[^ ^180^ ^]^ compound 13a and 13b (CRBN‐recruiting EGFR of L858R/T790M degraders),^[^ ^181^ ^]^ which can be further developed and evaluated in clinical studies as novel therapeutics to overcome EGFR‐TKI‐induced resistance. EGFR‐targeted PROTACs can selectively degrade mutant EGFR proteins without inhibiting EGFR^wt^, thus eliminating potential toxic side effects caused by the inhibition of EGFR^wt^. Given that PROTAC degrades proteins using the ubiquitin–proteasome system, it can not only eliminate enzyme activity but also the scaffolding function of the protein. This event‐driven pharmacological mechanism enables a single PROTAC molecule to mediate multiple rounds of target protein degradation and achieve therapeutic effects at a low drug concentration. However, due to the generally large molecular weight of PROTACs, it has poor solubility, low oral bioavailability, and poor membrane permeability, which pose challenges in EGFR‐targeting drug development. Expanding portfolio of PROTAC candidates will continue the promise of personalized and targeted therapy to improve the outcome of patients.^[^ ^182^ ^]^

Compared with PROTACs, MG molecules do not have a linker, making them a smaller molecular weight, which increases oral bioavailability and improves cellular permeability. Unlike PROTACs, which are heterobifunctional degraders simultaneously interacting with the E3 ligase and the targeted protein, MG degraders could interact with only the ligase (more frequently) or the targeted protein, and induce/stabilize their interactions. Two major categories of chemical compounds are aryl sulfonamides^[^ ^183^ ^]^ and thalidomide analogs.^[^ ^184^, ^185^ ^]^ However, the discovery of MG degraders often arises from serendipitous findings, and effective methods for their systematic design or identification are notably lacking. Therefore, genome‐wide CRISPR‐Cas9 knockout screening, phenotype‐based cytotoxicity assays, and large‐scale screening of clinically used drugs are being employed to identify compounds with MG‐like properties and assess their interactions with E3 ubiquitin ligases.^[^ ^186^, ^187^, ^188^ ^]^ However, research on MG targeting EGFR is currently very limited. It was discovered a new MG bardoxolone methyl (CDDO‐Me) directly bind to the tyrosine kinase domain of EGFR. It promotes EGFR's interaction with kelch‐like ECH‐associated protein 1 (KEAP1), an E3 ubiquitin ligase adapter, which induces ubiquitination of K63 ligation, and degrades EGFR through the autophagy‐lysosomal pathway. It also exhibits good anti‐tumor effects in the organoids derived from patients with triple‐negative breast cancer.^[^ ^189^ ^]^ The ongoing exploration of the potential of PROTACs and molecular glues will open exciting avenues for future research on targeting EGFR mutants.

### 3.3 Clinical Data of ICBs in EGFR^mut^ and EGFR^wt^ Patients

In this part, we summarized the clinical efficacy of ICBs monotherapy or ICB‐based combination therapy in EGFR^mut^ and EGFR^wt^ patients (**Table** **3**). Overall, EGFR^wt^ patients consistently derive greater benefits from ICBs than EGFR^mut^ patients in the following four types of treatments. In a large retrospective analysis of ICBs monotherapy or multiple ICBs, EGFR^wt^ patients represent a better response rate. Although the prognosis of EGFR^mut^ patients is slightly better in some individual cohorts, the overall response rate is quite low. The efficacy of the L858R and rare mutation subgroups is slightly better than that of the Ex19Del subgroup. The efficacy of EGFR^wt^ patients still prevails in ICBs combined with chemotherapy. However, in the EGFR^mut^ patient subgroups of L858R, T790M‐negative and TKI‐naïve, there is a certain improvement in clinical benefit compared with ICBs monotherapy. In a group of ICBs combined with TKIs, although EGFR^mut^ patients show antitumor activity, severe toxicities (such as liver injury, interstitial lung disease) limit clinical application. Mutant patients who have not received TKI respond relatively well, but safety issues overshadow the overall benefit, and there is no significant advantage compared with wild‐type patients.

**Table 3:** Clinical outcome of ICBs in cancer patients harboring EGFR^wt^ and EGFR^mut^.

Name of study	Phase	No. of EGFR (+)	Treatment	Treatment line	ORR	Median PFS	Median OS	Ref
Monotherapy								
KEYNOTE001	1	30	Pembrolizumab	First‐line	TKI naïve: 50% TKI pre‐treated: 4%	TKI naïve: 157.5 d TKI pre‐treated: 56 d	TKI naïve: 559 d TKI pre‐treated: 120 d	[190]
CheckMate012	1	**Mutant–7** (Ex19Del: 8 Exon 21 L858R: 3 Unknown: 1) **Wild‐type–30**	Nivolumab	First‐line	Mutant: 14% WT: 30%	Mutant: 1.8 mo WT: 6.6 mo	Mutant: 18.8 mo (0.2, 19.6+) WT: NR (1.8, 35.8+)	[448]
NCT02879994	2	**Mutant–10** (Ex19Del: 3 Exon 21 L858R: 4 Exon 20 insertion: 2 E330K: 1)	Pembrolizumab	First‐line	0%	119d	–	[190]
POPLAR	2	**Mutant—19** **Wild‐type—147**	Atezolizumab	Second or subsequent	NA	NA	PD‐(L)1 inhibitor vs docetaxel Mutant: HR 0.99 (0.29, 3.40) WT: HR 0.70(0.47, 1.04)	[205, 449]
BIRCH	2	**Mutant—13** **Wild‐type—104**	Atezolizumab		Mutant: 31% WT: 22%	NA	Mutant: 26 mo WT: 20.1 mo	[450]
KEYNOTE010	2/3	**Mutant–86** **Wild‐type–875**	Pembrolizumab	Second or subsequent	NA	Mutant: HR 1.79(0.94, 3.42) WT: HR 0.83(0.71, 0.98)	PD‐(L)1 inhibitor vs docetaxel Mutant: HR 0.88 (0.45, 1.70) WT: HR 0.66(0.55, 0.80)	[451]
OAK	3	**Mutant–85** **Wild‐type–628**	Atezolizumab	Second or subsequent	NA	NA	PD‐(L)1 inhibitor vs docetaxel Mutant: HR 1.24 (0.71, 2.18) WT: HR 0.69(0.57, 0.83)	[452]
Checkmate 057	3	**Mutant–8** **Not detected–340**	Nivolumab	Second or subsequent	11%	HR 1.46 (0.90, 2.37) Not detected: HR 0.83(0.65, 1.06)	PD‐(L)1 inhibitor vs docetaxel Mutant: HR 1.18 (0.69, 2.00) Not detected: HR 0.66(0.51, 0.86)	[453]
Multiple ICB therapy								
CheckMate012	1	**Mutant–8** **Wild‐type–54**	Nivolumab + ipilimumab	First‐line	Mutant: 50% WT: 41%	–	–	[194]
KEYNOTE021 Cohort D+H	1/2	**Mutant–10** **Wild‐type–33**	Pembrolizumab + ipilimumab	Second or subsequent	Mutant: 10% WT: NA	–	–	[454]
Combined with chemotherapy								
CheckMate012	1	**Mutant–6** **Wild‐type–30**	Nivolumab + chemotherapy	First‐line	Mutant: 17% WT: 47%	Mutant: 4.8 mo WT: 7.5 mo	Mutant: 20.5 mo WT: 24.5 mo	[196]
NCT03513666	2	**Mutant—40** (Ex19Del: 23 Exon 21 L858R: 17)	Toripalimab + chemotherapy	Second or subsequent	Mutant: 50%	Mutant: 7.0 mo	–	[455]
NCT03924050	2	**Mutant—40** (Ex19 Del: 23 Exon 21 L858R: 17)	Toripalimab + chemotherapy	Second or subsequent	Mutant: (Ex19 Del: 58.8% L858R: 43.5%)	Mutant (Ex19Del: 5.4 mo L858R: 7.6 mo)	Mutant (Ex19Del: 18 mo L858R: 23.5 mo)	[456]
PROLUNG	2	**EGFR/ALK mutant–25** **Wild‐type–53**	Pembrolizumab + chemotherapy	Second or subsequent	Mutant: 58.3% WT: 35.7%	Mutant: 6.8 mo WT: 9.5 mo	Mutant: 4.6 mo (0.83–26.2) WT: 4.1 mo (0.97–17.1)	[457]
CheckMate722	3	**Mutant–144**	Nivolumab + chemotherapy	Second or subsequent	–	Mutant: 5.6 mo (4.5–6.8)	Mutant: 19.4 mo (16.1–21.0)	[458]
KEYNOTE‐789	3	**T790 mutation** (Positive: 151 Negative: 227)	Pembrolizumab + chemotherapy	Second or subsequent	–	PD‐(L)1 inhibitor+ chemotherapy vs placebo+ chemotherapy Positive: HR 0.92 (0.67–1.27) Negative: HR 0.79 (0.61–1.03)	PD‐(L)1 inhibitor+ chemotherapy vs placebo+ chemotherapy Positive: HR 0.93 (0.69–1.26) Negative: HR 0.81 (0.62–1.04)	[459]
		**EGFR‐activating mutation** (L858R: 170 Ex19Del: 238)				L858R: HR 0.86 (0.64–1.17) Ex19Del: HR 0.82 (0.63–1.06)	L858R: HR 0.94 (0.70–1.26) Del9: HR 0.82 (0.64–1.05)	
Combined with EGFR TKIs								
CheckMate012	1	**Mutant—21** **Mutant, TKI‐naïve–20**	Nivolumab + erlotinib	First‐line Second or subsequent	Mutant: 19% Mutant, TKI‐naive: 15%	Mutant: 5.1 mo (2.3–12.1) Mutant, TKI‐naive: 16.6 mo	Mutant: 18.7 mo (7.3‐NA)	[414]
NCT02088112	1	**Mutant–40**	Durvalumab+ gefitinib	First‐line Second or subsequent	TKI naïve: 63.3% TKI pre‐treated: 70.0%	TKI naïve: 10.1 mo TKI pre‐treated: 12.0 mo	–	[198]
NCT01998126	1	**Mutant–11**	Ipilimumab + erlotinib	First‐line	–	17.9	>38	[460]
NCT02040064	1	**Mutant–27**	Tremelimumab+ gefitinib	Second	72%	2.2 (95%CI, 1.8–4.2)	–	[417]
TATTON	1b	**Mutant–23** (T790M positive: 13 T790M negative: 8) **Mutant, TKI‐naive–11**	Durvalumab+ osimertinib	Second or subsequent	Mutant: 43% (T790M positive: 25% T790M negative: 86%) Mutant, TKI‐naive: 70%	–	–	[199]
NCT02013219	1b	**Mutant–28**	Atezolizumab+ Erlotinib	First‐line	Mutant: 75%	Mutant: 11.3 mo	Mutant: 11.2 mo (0.8–24.2)	[461]
KEYNOTE021 Cohort E+F	1/2	**Mutant, TKI‐pretreated–12**	Pembrolizumab + erlotinib	First‐line	Mutant: 41.7%	Mutant: 19.5 mo	Mutant: NR (19.5‐NR)	[202]
		**Mutant–7**	Pembrolizumab + gefitinib	First‐line	Mutant: 14.3%	Mutant: 1.4 mo	Mutant: 13.0 mo (0.2‐NR)	
CAURAL	3B‐4	**Mutant—14** (T790M positive)	Durvalumab+ osimertinib	Second or subsequent	Mutant: 60%	–	–	[462]
Large retrospective analysis								
Italian EAP	–	**Mutant–102** **Wild‐type–1293**	Nivolumab	Second or subsequent	Mutant: 8.8% WT: 19.6%	Mutant: 3.0 mo (2.7–3.3) WT: 3.0 mo (2.8–3.1)	Mutant: 8.3 mo (2.2–14.4) WT: 11.0 mo (10.0–12.0)	[463]
Immunotarget	–	**Mutant–125**	Multiple ICBs	Across lines	Mutant: 12%	Mutant: 2.1 mo (1.8–2.7) (T790M: 1.4 Exon 19: 1.8 Exon 21: 2.5 Other mutations: 2.8)	Mutant: 10 mo (6.7–14.2)	[192]
CRD42019117020	–	–	Anti‐PD1/PD‐L1 inhibitors	Across lines	–	Mutant: HR 1.57 (1.07–2.31) WT: HR 0.83 (0.73–0.95)	Mutant: HR 1.11 (0.8–1.52) WT: HR 0.7 (0.63–0.77)	[464]
	–	**EGFR‐mutant or ALK‐positive—28** **EGFR WT/ALK‐negative–30**	Anti‐PD1/PD‐L1 inhibitors	–	Mutant: 3.6% WT: 23.3%	Mutant: 2.07 mo (1.84–2.07) WT: 2.58 mo (1.91–6.37)	–	[306]
	‐	**Mutant—171** (Ex19Del: 80 L858R: 46 20Inser: 28 G719: 7 L861Q: 5 Other: 5) **WT—212**	Anti‐PD1/PD‐L1, CTLA4 inhibitors	Across lines	**Mutant** (Ex19Del: 7% L858R: 16%) **WT—22%**	**Mutant—1.8 mo (0**–**40.5)** (Ex19Del: 1.6 L858R: 1.9 20Inser: 1.9 G719: 4.8 L861Q: 1.3 Other: 2.6)	**Mutant—9.4 mo (0.1**–**73.3)** (Ex19Del: 9.4 L858R: 12.1 20Inser: 5.5 G719: 29.0 L861Q: 5.2 Other: 11.4)	[191]
	‐	**Mutant–25** (T790M positive: 8 T790M negative: 17)	Nivolumab+ osimertinib	Second or subsequent	‐	**Mutant—1.5 mo (1.3**–**2.8)** T790M positive: 1.3 mo (0.1–1.8) T790M negative: 2.1 mo (1.3–3.4)	‐	[193]

HR, hazard ratio; mOS, median overall survival; mPFS, median progression‐free survival; NA, not applicable; NE, not estimable; ORR, overall response rate; PD‐1, programmed cell death protein‐1; PD‐L1, programmed death ligand‐1; TKI, tyrosine kinase inhibitor

#### 3.3.1 ICBs Monotherapy

Clinical benefit of ICBs therapy has been less frequently observed in EGFR^mut^ patients compared to EGFR^wt^ patients in either first‐line treatment (including KEYNOTE‐001,^[^ ^190^ ^]^ CheckMate012, NCT02879994) or in the second or subsequent line, including CheckMate057, OAK, POPLAR, and KEYNOTE‐010, with the criteria of objective response rate (ORR), median progression‐free survival (mPFS), or median overall survival (mOS). Similar results are also found in the large retrospective analysis,^[^ ^191^ ^]^ the ORR of ICBs was 12% and the PFS was only 2.1 months in patients with EGFR mutations,^[^ ^192^ ^]^ suggesting the efficacy of ICBs is minimal in EGFR^mut^ patients. Interestingly, responses to ICBs also vary depending on the EGFR mutation subtype.^[^ ^191^ ^]^ L858R group (16%) exhibits a more favorable response rate compared with Ex19Del subgroups (7%). Uncommon mutation subtypes such as G719X and L861Q, which account for ≈7% of all EGFR^mut^ NSCLC, are also associated with a more favorable response to ICBs. This can partly be explained by the differences in tumor mutation burden (TMB), as Ex19Del is associated with lower TMB levels than L858R mutation despite similar smoking history. Patients positive with T790M mutations display worse mPFS than patients negative with T790M mutations.^[^ ^193^ ^]^ These findings emphasize the importance of evaluating EGFR mutation subtypes for personalized ICB use.

#### 3.3.2 Multiple ICBs

When patients are treated with multiple ICBs such as anti‐PD1 plus anti‐CTLA4 blockade, there's no big clinical difference between EGFR^mut^ and EGFR^wt^ patients in CheckMate012,^[^ ^194^ ^]^ and in KEYNOTE021 cohort D and H, the objective response rate is quite low in EGFR^mut^ patients.^[^ ^195^ ^]^

#### 3.3.3 ICBs Combined with Chemotherapy

When patients were treated with combination therapy of ICBs and chemotherapy, although EGFR^mut^ patients showed less clinical benefit compared to EGFR^wt^ patients displaying shorter mPFS and mOS in CheckMate012,^[^ ^196^ ^]^ but this kind of combination therapy alleviates the disease progression of EGFR^mut^ patients to a certain extent, as shown in clinical trials including NCT03513666, CheckMate‐722, and KEYNOTE‐789. Similar to ICB monotherapy, different EGFR mutation subtypes also showed distinct responses. By stratified analysis for ICB‐based regimen versus chemotherapy alone, it showed that patients with EGFR L858R benefit most and patients with Ex19Del benefit least in the EGFR mutant groups. Patients without the T790M mutation and received one‐line TKI previously could benefit more from ICB‐based regimen. Patients with the L858R mutation and without the T790M mutation displayed a significantly greater benefit from ICB‐based regimens compared to their counterparts.^[^ ^197^ ^]^

#### 3.3.4 ICBs Combined with TKIs

In EGFR^mut^ subgroup, pembrolizumab functioned better in patients who were TKI‐naïve compared with those previously treated with TKI in the KEYNOTE‐001 trial.^[^ ^190^ ^]^ Next, we summarized the efficacy of ICBs plus EGFR TKIs. Although antitumor activity was noticed in the EGFR^mut^ patients treated with durvalumab plus gefitinib,^[^ ^198^ ^]^ but this combination also led to liver toxicity and subsequently a high discontinuation rate. Similar results were also noticed in the TATTON,^[^ ^199^ ^]^ which displayed the high incidence of interstitial lung diseases, thus leading to the stop of recruitment of the phase 3 trial named CAURAL.^[^ ^200^ ^]^ And in NCT02013219, EGFR^mut^ patients gained a good response to the combination of atezolizumab and erlotinib, but nearly 50% patients were associated with treatment‐related serious adverse events (AEs),^[^ ^201^ ^]^ and similar toxicity issues of high‐grade liver toxicity happened in the KEYNOTE021.^[^ ^202^ ^]^

In addition, the treatment sequence of ICBs and EGFR TKIs, and the combination with what kinds of EGFR TKIs may affect the incidence of treatment‐related toxicity. Previous results showed that ICBs functioned better in TKI‐naïve patients, suggesting TKI treatment altered the immune profiles, which we will describe in the next part. One study showed that patients pre‐treated with nivolumab, then followed by osimertinib, had serious AEs,^[^ ^203^ ^]^ but it was not noticed in the patients pre‐treated with anti‐PD(L)1 blockade, then followed by other EGFR TKIs (such as afatinib or erlotinib).^[^ ^204^ ^]^ Overall, ICBs cannot improve the overall survival of patients with EGFR mutations, but it brings significant survival benefits to the subgroup of patients with wild‐type EGFR.^[^ ^205^ ^]^ The benefits of EGFR^mut^ patients highly depend on the subtype (L858R > Ex19Del, T790M negative> T790 positive) and treatment background (such as TKI‐naïve), and the combination of ICB‐TKI further complicates the efficacy assessment due to toxicity issues. As a major regulator of the transition between immunosuppressive and immunostimulatory phenotypes, EGFR‐TKIs can enhance the efficacy of immunotherapy by inhibiting EGFR. It is necessary to explore the TME of EGFR‐related cancers, study its changes after TKI treatment, and the resulting downstream physiological changes. Jia et al. studied an orthotopic lung cancer mouse model driven by EGFR and found that treatment with EGFR‐TKIs could reduce tumor volume, accompanied by the infiltration of cytotoxic T lymphocytes (CTLs) and dendritic cells (DCs). They also found that treatment with osimertinib and gefitinib could overcome the immunosuppressive TME by inhibiting M2 polarization and depleting forkhead box protein P3 (Foxp3)^+^ regulatory T cells (Tregs). However, the level of myeloid‐derived suppressor cells (MDSCs) remained unchanged throughout the process, which also explains why the combination of EGFR‐TKIs and immunotherapeutic drugs has only moderate effects.^[^ ^206^ ^]^

## 4 Reasons of EGFR Activation and Mutation Affecting the Effectiveness of Immunotherapy

**Figure 4:**
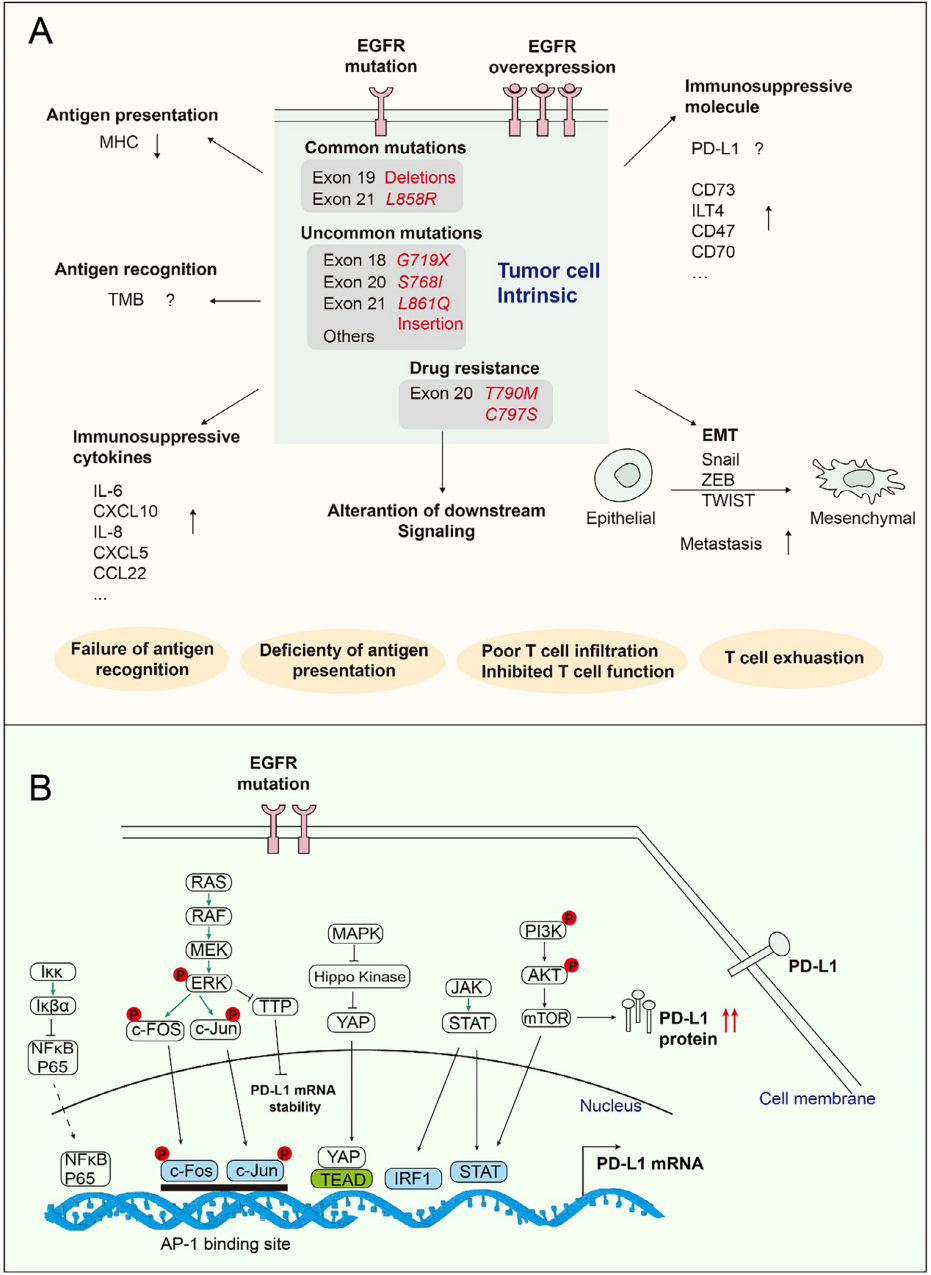
EGFR contributes to immunotherapy resistance by modulating multiple tumor‐intrinsic mechanisms, taking PD‐L1 regulation as an example. A) The impact of EGFR on tumor cells. Activated and mutated EGFR in tumor cells affect immunotherapy in four ways: 1) EGFR signaling decreases MHC levels in tumor cells, reducing their antigen presentation; 2) EGFR modulates TMB levels in tumor cells, reducing their recognition by immune cells; 3) EGFR signaling promotes the expression of immune‐suppressive molecules like CD73 and ILT4 in tumor cells, suppressing immune cell function; 4) EGFR signaling stimulates tumor cells to secrete immunosuppressive cytokines such as IL‐6 and CXCL10, inhibiting immune cell infiltration and function; 5) EGFR signaling can alter tumor cell morphology, inducing mesenchymal transition and promoting metastasis. B) PD‐L1 expression is regulated by the downstream of EGFR mutation.  EGFR mutation stimulates PD‐L1 transcription levels via multiple downstream oncogenic signaling pathways, enhances the mRNA stability via the RAS‐ERK1/2‐TPP pathway, and enhances PD‐L1 protein stability via the PI3K/mTOR pathway.

We integrated several parameters from a tumor‐intrinsic and tumor‐extrinsic perspective to explain how EGFR activation and mutation affect the effectiveness of immunotherapy. Activated and mutated EGFR in tumor cells modulates tumor‐intrinsic mechanisms mainly in four ways, which are discussed below and illustrated in **Figure** **4**A,B.

### 4.1 Tumor Intrinsic

#### 4.1.1 Antigen Presentation and Recognition

##### Expression of Major Histocompatibility Complex (MHC)

EGFR can combat the immune response by modulating the expression of numerous molecules involved in antigen presentation, such as MHC, or known as human leukocyte antigen (HLA) in humans. These molecules reside on the cell surface and are capable of recognizing and binding external antigens. Once an antigen is bound to an MHC molecule, an MHC‐antigen complex is formed, which can be recognized by specific T cells, thereby triggering the immune response. There are two principal types of MHC molecules: MHC‐I and MHC‐II. MHC‐I molecules are ubiquitously expressed on various cell surfaces and are primarily responsible for presenting endogenous antigens. In contrast, MHC‐II molecules are predominantly found on the antigen‐presenting cells (APCs), including macrophages, DCs, and B cells. Its primary function is to present exogenous antigens.^[^ ^207^ ^]^

Compared with EGFR/KRAS wild‐type tumors, EGFR^mut^ tumors had lower numbers of predicted MHC‐I and MHC‐II neoantigens.^[^ ^208^ ^]^ Studies focusing on the EGFR activation and MHC molecules of tumor cells have indicated that downstream PI3K/AKT,^[^ ^209^ ^]^ MAPK/ERK pathways ^[^ ^210^ ^]^ effectively reduce both MHC‐I and MHC‐II expression. EGFR inhibition using both TKIs and ligand‐blocking antibody (cetuximab) can also augment MHC‐I and MHC‐II expression, potentially via its effect on the interferons (IFN)‐γ receptor complex, class II major histocompatibility complex transactivator (CIITA) mRNA, and/or direct effect on the promoter of MHC genes.^[^ ^211^ ^]^ But it was also reported that in EGFR^mut^ lung adenocarcinoma, MHC‐I genes were downregulated to evade CD8^+^ T cells, and the level of MHC class II molecules was enhanced to engage with CD4^+^ Tregs^[^ ^212^ ^]^ and confer suppressive functions to Tregs.^[^ ^213^ ^]^

##### TMB

TMB refers to the number of non‐synonymous mutations in somatic cells within a specific genomic region, which can indirectly reflect the ability and degree of tumor production of new antigens, emerging as a biomarker for predicting the prognosis after ICB treatment.^[^ ^214^ ^]^ Compared with wild‐type tumors, EGFR^mut^ tumors exhibit lower TMB and reduced production of neoantigens, less clonal expansion, and show less response to ICBs.^[^ ^215^, ^216^ ^]^ Across all *EGFR* mutation subtypes, the median TMB in EGFR^mut^ patients was 3.8 non‐synonymous mutations/ megabase (Mb), much lower than that of wild‐type patients 7.4 non‐synonymous mutations/Mb, and patients with EGFR Ex19Del mutations showed lower TMB than those with EGFR L858R mutations,^[^ ^191^ ^]^ which have a worse prognosis and aggressive phenotype.^[^ ^217^ ^]^ Compared with L858R‐mutant tumors, Ex19Del‐mutant tumors were infiltrated with less CD8^+^ PD‐1^+^ exhausted T cells and benefited less from ICBs. Interestingly, sensitive EGFR mutations (L858R/Ex19del) are associated with significantly elevated TMB compared to WT tumors.^[^ ^218^ ^]^ And compared with wild‐type, tumors with EGFR L858R mutation exhibit higher TMB,^[^ ^218^ ^]^ but contradictory findings suggest that EGFR L858R mutations may actually have the lowest TMB among EGFR mutant subtypes.^[^ ^219^ ^]^

Regarding this phenomenon, using comprehensive proteogenomic analysis of early‐stage NSCLC in a cohort of mainly non‐smokers, one potential explanation is EGFR^mut^ patients exhibit lower apolipoprotein B mRNA editing enzyme catalytic subunit (APOBEC) mutation characteristics in the early stage, which utilizes its deaminase activity to catalyze mRNA or DNA conversion from cytosine nucleotides to uracil, or cytosine nucleotides to thymine nucleotides by binding to RNA and/or DNA.^[^ ^220^ ^]^ However, it is also reported that TMB is not associated with the response of ICBs if driver mutations were present,^[^ ^221^ ^]^ and a uniform standard for the detection, calculation method, and cutoff values of TMB is needed. Therefore, the relationship between low TMB and poor response to ICBs in EGFR^mut^ patients should be further explored.

#### 4.1.2 Expression of Immune Suppression‐Related Molecules

##### PD‐1, PD‐L1

EGFR also affects the expression of some immunosuppressive molecules in tumor cells, among which the most studied is PD‐L1. PD‐L1 of tumor cells inhibits the function of T cells by binding to the PD‐1 receptor of T cells, thereby inhibiting the immune response. High PD‐L1 expression is more frequently observed in EGFR^mut^ than in wild‐type tumors,^[^ ^222^, ^223^, ^224^, ^225^ ^]^ which facilitates tumor immune evasion via activation of PD‐1 pathway and causes T‐cell apoptosis in vitro. But EGFR mutation raised the expression of PD‐L1 through diverse signaling pathways, including PI3K/Akt/mTOR,^[^ ^226^ ^]^ yes‐associated protein (YAP).^[^ ^227^ ^]^ NSCLC with mutant EGFR increases PD‐L1 expression by activating PI3K‐AKT and MEK‐ERK signaling pathways,^[^ ^228^ ^]^ or JAK/STAT.^[^ ^229^ ^]^ EGFR mutation can inhibit the activity of interferon regulatory factor 1 (IRF1) through PI3K, reducing the level of PD‐L1 by IFNγ.^[^ ^230^ ^]^ And ligand‐ or mutation‐activated EGFRs can stabilize PD‐L1 transcripts, together with EGFR‐ and PD‐L1‐dependent activation of phospholipase C (PLC)‐γ1, forming a positive feedback loop of oncogenic function of EGFR.^[^ ^231^ ^]^ EGFR‐L858R and Ex19Del mutation upregulated PD‐L1 via p‐ERK1/2/p‐c‐Jun,^[^ ^232^ ^]^ and EGFR‐T790M induced PD‐L1 via the nuclear factor kappa‐B (NF‐κB) signaling pathway.^[^ ^233^ ^]^

And EGFR‐TKI could not only inhibit tumor cell viability but also enhance antitumor immunity via reducing PD‐L1 expression level,^[^ ^232^ ^]^ for example, gefitinib can reduce PD‐L1 expression via inhibiting NF‐κB signaling in EGFR^mut^ tumors.^[^ ^234^ ^]^ Therefore, EGFR activation promotes PD‐L1 expression via NF‐κB signaling. Currently, experimental data suggest that EGFR mutations could upregulate PD‐L1 expression by means of five possible pathways, such as NF‐kB, Ras/RAF/MEK/ERK, YAP, JAK/STAT, and PI3K/AKT/mTOR (Figure 4B). The epidemiologic relationship between EGFR mutations and PD‐L1 expression still remains controversial. Some studies showed that EGFR^mut^ patients have decreased PD‐L1 expression,^[^ ^216^, ^235^, ^236^ ^]^ so evaluating PD‐L1 expression as a response rate of EGFR^wt^ and EGFR^mut^ patients to ICBs is not an ideal choice.

##### Others (CD73, ILT4, CD47, CD70, LAG3)

Except for PD‐L1, CD73 (also known as ecto‐5′‐nucleotidase) is also a novel immune checkpoint associated with catalyzing the formation of extracellular adenosine from adenosine 5′‐monophosphate (AMP) on the cell surface.^[^ ^237^, ^238^ ^]^ EGFR^mut^ NSCLC tumors have higher expression of CD73 compared to wild‐type EGFR,^[^ ^239^, ^240^ ^]^ which is regulated by the Ras‐Raf‐ERK pathway^[^ ^241^ ^]^ and ERK‐JUN pathway.^[^ ^242^ ^]^ Elevated CD73 expression may serve as a predictor of ICBs efficacy in EGFR^mut^ patients, whereas it does not significantly impact treatment outcomes in those without EGFR mutations.^[^ ^243^ ^]^ On the basis of these findings, the combination of anti‐CD73 antibody named oleclumab (MEDI9447) and anti‐PD‐L1 antibody named durvalumab has been suggested to be effective against EGFR^mut^ lung cancer.^[^ ^239^ ^]^

Immunoglobulin‐like transcript 4 (ILT4) is another immune inhibitory receptor in the ILT superfamily, which is mainly expressed on myeloid cells, but it is also highly expressed on tumor cells.^[^ ^244^ ^]^ In EGFR^mut^ tumors, ILT4 was upregulated through the EGFR‐AKT‐ERK1/2 signaling, which induced recruitment and M2‐like polarization of TAMs, meanwhile blocked T cell infiltration and cytotoxicity, thereby promoting the formation of an immunosuppressive microenvironment.^[^ ^245^ ^]^

CD47 (also known as integrin‐associated protein, IAP) is a novel immune checkpoint that interacts with signal regulatory protein alpha (SIRPα) to deliver a “don't eat me” signal, allowing cancer cells to avoid elimination by phagocytes and diminish the initiation of adaptive immune responses.^[^ ^246^, ^247^ ^]^ It has been reported that EGFR TKI resistance is associated with high expression of CD47, blockade of CD47 would enhance the antitumor efficacy of osimertinib.^[^ ^248^, ^249^ ^]^ Lyu et.al found that EGFR activation with EGF ligand and ectopically expressed EGFRvIII mutant induces CD47 protein upregulation via enhancing the binding of c‐Src to CD47, resulting c‐Src phosphorylated CD47 at Y288.^[^ ^250^ ^]^ Interestingly, through screening immune checkpoint‐related genes in clinical samples and NSCLC cell line models, CD47 was found to be the most relevant to EGFR activation. Activating EGFR mutations could transcriptionally increase CD47 expression via the activation of ERK/c‐Myc and AKT/NF‐κB signaling, which impairs macrophage phagocytosis and mediates innate immune evasion of cancer cells.^[^ ^251^ ^]^

CD70, a member of the tumor necrosis factor receptor (TNFR) superfamily, is the unique ligand of CD27. Normally, it is mainly expressed on the lymphocytes, but recent research suggests it is also expressed on many cancer cells. Under normal conditions, CD70/CD27 axis could promote T cell activation and proliferation by activating NFκB and c‐Jun kinase pathways. But some cancer cells overexpressed CD70, leading to chronic co‐stimulation and the expression of immune checkpoints such as PD‐1 and TIM‐3 on T cells, resulting in immune exhaustion.^[^ ^252^, ^253^ ^]^ John et al. found that CD70 is upregulated on EGFR^mut^ NSCLC cells, which is under acquired, epithelial to mesenchymal transition ‐associated EGFR TKI resistance. In the clinical trial, among 39 patients with EGFR TKI‐refractory NSCLC, 33 patients with high CD70 expression had a higher risk of death (4.95‐fold) compared with the low CD70 group.^[^ ^254^ ^]^

TIM‐3 (also known as HAVCR2) is a co‐inhibitory receptor immune checkpoint protein, which was initially identified as expressed exclusively on the surface of differentiated T helper 1 (Th1) cells, IFNγ‐producing CD8^+^ T cells, and NK cells,^[^ ^255^ ^]^ but it was also identified on lung cancer.^[^ ^256^ ^]^ Compared with tumors lacking mutations in both KRAS and EGFR, EGFR^mut^ lung adenocarcinomas had significantly lower TIM‐3.^[^ ^256^ ^]^

Another inhibitory receptor, LAG‐3 is highly expressed in exhausted T cells. Some studies showed there is no association of LAG‐3 with *EGFR* status in two prior studies,^[^ ^256^, ^257^ ^]^ lower LAG‐3 expression in *EGFR*‐mutated adenocarcinomas, which is expected given their lower immunogenicity as compared to *EGFR*‐wild‐type counterparts.^[^ ^258^ ^]^

#### 4.1.3 Morphological Changes, Such as EMT/EMP

Tumor epithelial‐to‐mesenchymal transition/plasticity (EMT/EMP) also accounts for the immunotherapy resistance in cancer.^[^ ^259^ ^]^ Accumulating evidence showed EGFR signaling drives tumor EMT,^[^ ^260^, ^261^ ^]^ and cancer cells with long exposure to EGFR‐TKIs acquired mesenchymal properties,^[^ ^262^, ^263^, ^264^, ^265^ ^]^ which decreased tumor lysis in response to immune effector mechanisms.^[^ ^266^ ^]^ Using liquid biopsy, it is found that higher mesenchymal/EMT markers (Vimentin, TWIST‐1, AXL) are expressed in circulating tumor cells (CTCs) of EGFR^mut^ NSCLC patients under osimertinib, who developed at progression of disease with higher PD‐L1 expression level.^[^ ^267^ ^]^ EGFR regulates the expression of 171 EMT‐related genes through the MAPK pathway,^[^ ^268^ ^]^ and EGFR activation and mutants can stimulate EMT via snail2,^[^ ^269^ ^]^ zeb1,^[^ ^270^ ^]^ twist,^[^ ^261^ ^]^ thus, tumor cells undergoing EMT form an immunosuppressive microenvironment and have a poor response to immunotherapy.^[^ ^271^ ^]^

#### 4.1.4 Release of Immunosuppressive Cytokines

Cytokines are essential components of TME, which serve as mediators of cell‐to‐cell signaling that control the homeostasis of TME, including interleukins, interferon, members of the tumor necrosis factor superfamily, colony‐stimulating factors, chemokines, and growth factors.^[^ ^272^, ^273^ ^]^ Next, we mainly focused on the currently reported cytokines function in the EGFR^mut^ and EGFR^wt^ formed TME, which secretes multiple negative immune regulatory factors, inhibiting the recognition and presentation of tumor antigens and the antitumor effect of immune cells, resulting in immune escape.

Vascular endothelial growth factor (VEGF) is a key regulator of pathological angiogenesis associated with tumors and recruits immunosuppressive cells.^[^ ^274^ ^]^ It was reported that EGFR^mut^ tumors have higher levels of VEGF than wild‐type lung tumors.^[^ ^275^ ^]^ EGFR activation can drive VEGF expression via upregulating hypoxia‐inducible factor‐1 (HIF1)α in a hypoxia‐independent way,^[^ ^276^ ^]^ EGFR inhibition using ZD1839 leads to lower VEGF.^[^ ^277^ ^]^ A meta‐analysis of a total of 1772 EGFR^mut^ NSCLC patients showed combination of VEGF inhibition and anti‐PD(L)1 had better clinical outcomes compared with using PD(L)1 inhibitor only,^[^ ^278^ ^]^ supporting the addition of VEGF inhibitor to immunotherapy or even chemotherapy could be the preferred option for TKI‐resistance and EGFR^mut^ NSCLC.^[^ ^197^ ^]^

Interleukin‐6 (IL6) acts as both a pro‐inflammatory and an anti‐inflammatory cytokine,^[^ ^279^ ^]^ IL6 expression was higher in ICB‐induced immune‐related enterocolitis. Blockade of IL6 abrogates immunotherapy toxicity and promotes tumor immunity, showing a higher density of CD4^+^/CD8^+^ effector T cells.^[^ ^280^ ^]^ Ovarian cancer cells overexpressing EGFR enhanced IL6 production and secretion by activating the JAK2/STAT3 signaling.^[^ ^281^ ^]^ In EGFR^mut^ mouse model (L858R or T790M) and EGFR‐transfected primary human endobronchial cells, IL6 expression was elevated.^[^ ^282^ ^]^ A similar phenotype was found in the EGFR^mut^ NSCLC tumors with acquired EGFR‐TKI resistance; IL6 is upregulated.^[^ ^283^ ^]^

TNF can promote T‐cell adhesion and recruit immunosuppressive cells, such as Tregs, and impair infiltration of CD8^+^ effector T cells.^[^ ^284^ ^]^ EGFR activation can modulate higher miR‐21 expression, which decreases the TNF level by impairing the stability of TNF mRNA.^[^ ^285^ ^]^

C‐X‐C motif chemokine ligand 10 (CXCL10) is a member of the CXC chemokine family and a potent chemoattractant for activated T cells, NK cells, and monocytes,^[^ ^286^ ^]^ and is a positive prognostic factor for response to immunotherapy in melanoma and is associated with an increasing number of CD8^+^ T cells.^[^ ^287^ ^]^ In EGFR‐mutant lung cancer with EGFR‐TKI treatment, CXCL10 level was found to be elevated,^[^ ^230^, ^288^ ^]^ and EGFR signaling suppressed the CXCL10 expression via inducing histone deacetylation.^[^ ^289^ ^]^ Mechanistically, EGFR signaling reduced CXCL10 via reducing activity of IRF1,^[^ ^230^ ^]^ and CXCL10 activates chemokine receptor 3 (CXCR3) expressed on B and T lymphocytes, NK cells, DCs, and nonhematopoietic cells.^[^ ^290^ ^]^

CXCL5 is another member of the CXC chemokine family, and CXCR2 is its specific receptor with granulocyte chemotactic activity. It also has functions such as promoting angiogenesis and mediating inflammatory responses,^[^ ^291^ ^]^ an independent predictor for unfavorable response to ICBs.^[^ ^292^ ^]^ In the EGFR‐negative group, CXCL5 was highly expressed.^[^ ^293^ ^]^ Mechanistically, CXCL5 overexpression promotes immune escape via PD‐L1 upregulation in the cascade of Paxillin/AKT signaling.^[^ ^294^ ^]^ EGFR promotes the expression of CXCL5 in cancer cells through the ERK1/2, PI3K/Akt, and p38 MAPK signaling pathways, thereby recruiting leucocytes, such as monocytes, neutrophils recruited into the TME to form an inflammatory microenvironment, which can promote the metastasis and development of cancer cells.^[^ ^295^, ^296^ ^]^

IL‐8, also known as chemokine CXCL8, is a chemokine containing the ELR (glutamate leucine arginine) sequence that can promote tumor cell stemness and metastasis. Higher levels of serum IL8 were associated with poorer response to ICBs.^[^ ^297^, ^298^ ^]^ And it can promote the transfer of these immunosuppressive cells to the TME by binding to CXCR1 and CXCR2 of myeloid cells such as neutrophils.^[^ ^299^ ^]^ In EGFR^mut^ cells with gefitinib‐resistant^[^ ^300^ ^]^ and acquired resistance to erlotinib,^[^ ^266^ ^]^ IL‐8 was upregulated. In EGFR^mut^ tumors, IL8 expression was increased via activating PI3K/AKT/ERK signaling.^[^ ^301^, ^302^ ^]^

C–C motif chemokine ligand 22 (CCL22) is a member of the CC chemokine family. CCL22 can be secreted by dendritic cells and macrophages, and there are also studies indicating that CCL22 can be expressed by cancer cells. CCL22 regulates Treg migration via binding to its receptor CCR4.^[^ ^303^ ^]^ In EGFR^mut^ lung adenocarcinoma, EGFR signaling upregulates the expression of CCL22 by activating cJun/cJun N‐terminal kinase, thereby promoting tumor cell recruitment of Tregs and forming an immunosuppressive microenvironment.^[^ ^212^, ^230^ ^]^ EGFR activation was also found to promote the expression of CCL22 in human immortalized keratinocytes (HaCaT).^[^ ^304^ ^]^

In summary, the above studies indicate that after the activation of EGFR signaling in cancer cells, the recruitment of immune active cells is mainly reduced, and the recruitment of immune suppressive cells is increased by releasing cytokines to form an immune suppressive microenvironment, thereby promoting tumor immune escape.

### 4.2 Tumor Extrinsic

The concept of TME is persistently updating, the conventional refers to surrounding microenvironment in which tumor cells exist, recruiting non‐transformed cells such as stromal cells, immune cells, fibroblast accompanied by the release of diverse soluble molecules (cytokines, chemokines and vesicles), now it also concludes the peripheral microenvironment with distally located lymphatic tissues and even tumor organismal environment.^[^ ^305^ ^]^

#### 4.2.1 Overexpression and Mutation of EGFR Affects the TMEs

Tumors carrying EGFR mutations form an immunosuppressive microenvironment with immunologic tolerance and weak immunogenicity that protects them from the immune system attacking.^[^ ^216^, ^306^ ^]^ Higher infiltration of CD4^+^ T cells, neutrophils, macrophages, and DCs was found in the EGFR^mut^ glioma cases, which correlated with worse prognosis and shorter survival.^[^ ^307^ ^]^ Kim et al. compared single‐cell transcriptomic data from NSCLC patients with EGFR^mut^ and EGFR^wt^, which showed reduced CXCL13‐producing follicular helper CD4^+^ T(TFH)‐like cells and tissue‐resident memory CD8^+^ T(TRM)‐like cells.^[^ ^308^ ^]^ Using single‐cell RNA (sc‐RNA) and bulk RNA sequencing datasets of lung adenocarcinoma, CD8^+^ T cells and NK cells were found to decrease in EGFR^mut^ samples relative to EGFR^wt^ samples, whereas Tregs and myeloid cells showed an inverse tendency.^[^ ^212^ ^]^ Tumor‐infiltrating lymphocytes (TILs) are often sparse or absent in EGFR^mut^ tumors, resulting in impaired CD8^+^ T cell function. This is primarily attributed to the presence of bystander CD39^−^ CD8^+^ T cells, which lack cytotoxic activity, and the absence of IL‐10, which is essential for inducing CD39 expression on CD8^+^ T cells with cytotoxic and depletion characteristics. These mechanisms may help explain the adverse reactions to anti‐PD(L)1 therapy.^[^ ^309^ ^]^

Specifically, EGFR L858R expression rapidly induces CCL5^[^ ^310^ ^]^ in tumor cells, contributing to CD8^+^ T cell depletion and Tregs‐induced immunosuppressive environments.^[^ ^219^, ^310^ ^]^ The immune microenvironment of EGFR exon 20 insertion is more inhibitory than that of L858R and wild‐type, characterized by reduced infiltration of CD8^+^ FOXP3^−^ T cells in the tumor region and more M2‐like macrophages, while M1‐like macrophages produce fewer antitumor cytokines.^[^ ^311^ ^]^ Conversely, studies have shown that tumors with EGFR mutations (particularly L858R) exhibit higher CD3^+^ cell density in the tumor center and tumor infiltration margin, and tumors with sensitive EGFR mutation (L858R/Ex19del) are associated with a higher proportion of inflammatory immune phenotypes.^[^ ^218^ ^]^ As for EGFR‐T790M mutation, it has been demonstrated to induce immune escape mechanisms, characterized by reduced MHC‐I expression and increased expression of immune checkpoint molecules such as TIM‐3 and LAG‐3.^[^ ^312^ ^]^ In contrast, another study found that the T790M^−^ cohort exhibited a high T‐cell inflammatory gene expression profile score, PD‐L1 overexpression, and a chemokine‐rich immunosuppressive microenvironment, which was associated with significantly shortened patient progression time.^[^ ^313^ ^]^ As for the uncommon EGFR mutations (G719X, L861Q, S768I, and exon 20 insertions), they are associated with higher levels of CD8^+^ T cells and PD‐L1 expression compared with common types (Ex19Del and L858R),^[^ ^314^, ^315^ ^]^ which may benefit more from the immunotherapy.

**Figure 5:**
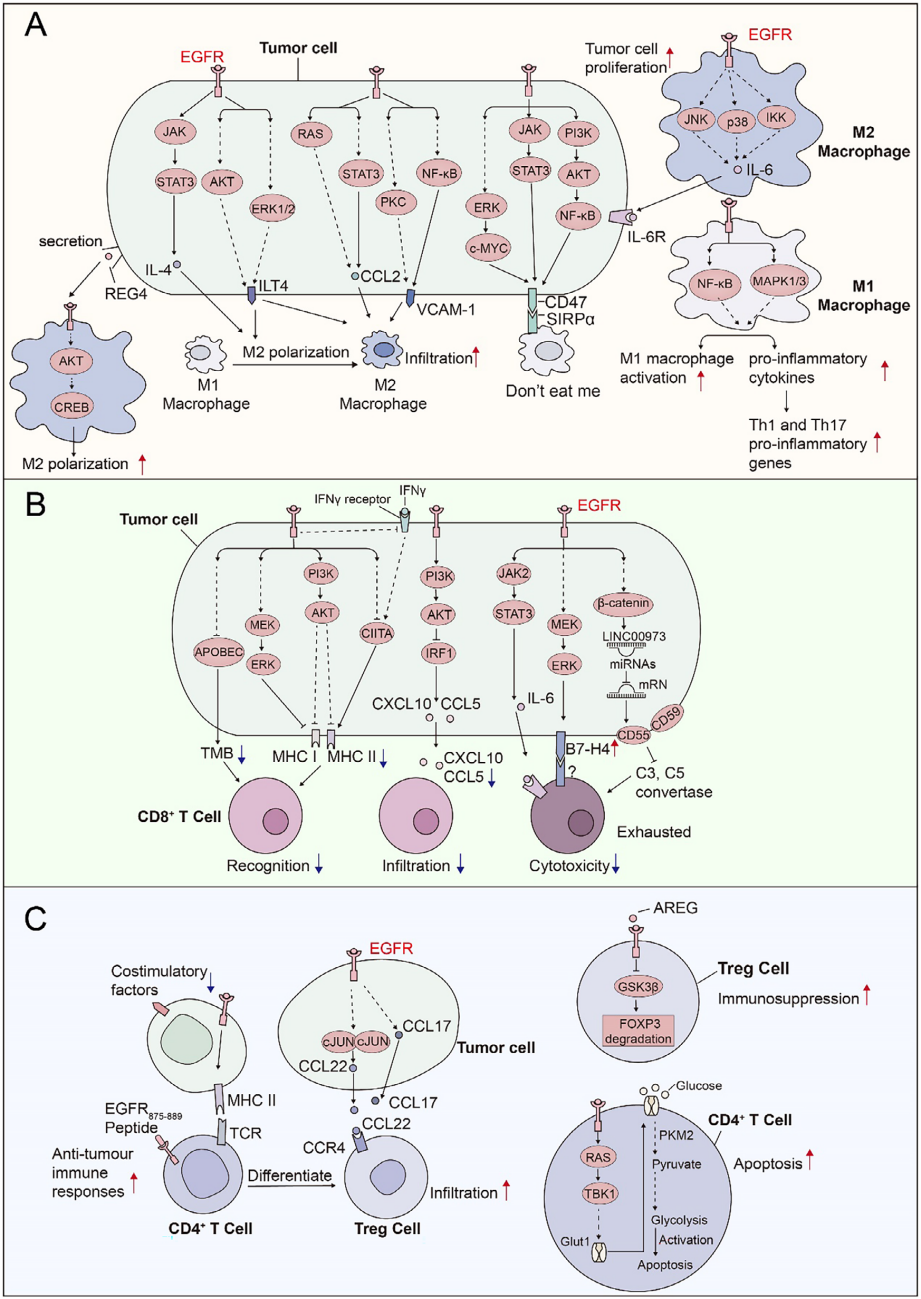
The activation and mutation of EGFR affect the efficacy of immunotherapy by influencing tumor cells and immune cells. A) EGFR is expressed in both tumor cells and macrophages. The EGFR signaling pathway in tumor cells affects macrophages in three ways: 1) Macrophage polarization: Tumor cell EGFR signaling promotes IL‐4 expression via the JAK‐STAT3 pathway, and ILT4 expression via the AKT and ERK1/2 pathways, driving the polarization of M1 macrophages into M2 macrophages; 2) Macrophage infiltration: ILT4 can enhance macrophage infiltration. In addition, tumor cells with EGFR signaling promote CCL2 expression through RAS and STAT3, and VCAM‐1 expression through PKC and NF‐κ B, facilitating macrophage infiltration into tumor cells; 3) Macrophage recognition: Tumor cells with EGFR signaling promote CD47 expression through the ERK‐c‐MYC, JAK‐STAT3, and PI3K/ AKT/ NF‐κ B signaling pathways. CD47 binds to macrophage SIRPα, releasing a "Don't eat me" signal that enables tumor cells to be recognized as self by macrophages and evade phagocytosis. The EGFR signaling in macrophages exerts multiple effects: 1) Macrophage polarization: Tumor‐cell‐secreted REG4 activates the EGFR‐AKT‐CREB pathway in macrophages, promoting M2 polarization; 2) Impacts on tumor cells: EGFR signaling in macrophage upregulates the expression and secretion of IL‐6 via the JNK, p38, and IKK pathways. IL‐6 then binds to the IL‐6 receptor on tumor cells, driving tumor cell proliferation; 3) Effects on other immune cells: EGFR signaling in macrophage promotes M1 macrophage activation through the NF‐κB and MAPK1/3 signaling pathways. It further leads to the secretion of pro‐inflammatory cytokines, which enhances the expression of Th1 and Th17 pro‐inflammatory genes. B) The impact of EGFR on CD8^+^ T cells. The EGFR signaling in tumor cells affects CD8^+^ T cells in three main ways: 1) Recognition by CD8^+^ T cells: Through EGFR signaling, tumor cells reduce APOBEC expression, lowering the TMB. Additionally, MHC I expression is inhibited by the MEK‐ERK pathway, and both MHC I and MHC II expression are suppressed by the PI3K‐AKT pathway, or via inhibiting the expression of CIITA. Tumor cells reduce their probability of being recognized by CD8^+^ T cells by decreasing TMB and MHC I/ II levels via modulating EGFR signaling; 2) CD8^+^ T cell infiltration: Tumor cells inhibit IRF1 expression through the EGFR‐PI3K‐AKT pathway, thereby suppressing the expression of CXCL10 and CCL5 and decreasing CD8^+^ T cell infiltration; 3) Cytotoxicity of CD8^+^ T cells: Tumor cells promote IL‐6 expression through the EGFR‐JAK2‐STAT3 pathway, and increase B7‐H4 expression through the MEK‐ERK pathway, and upregulate LINC00973 expression through β‐catenin. LINC00973 binds to miRNAs, protecting CD55 and CD59 mRNA from cleavage and enhancing their expression. CD55 and CD59 can decrease the secretion of cytokines essential for CD8^+^ T cell activation. C) The impact of EGFR on CD4^+^ T cells. EGFR is expressed in both tumor cells and CD4^+^ T cells. First, EGFR can act as an antigen, triggering an anti‐tumor immune response in CD4^+^ T cells; Second, tumor cell EGFR signaling promotes Treg cell infiltration. Tumor cells secrete CCL22 and CCL17, which bind to CCR4 on Tregs. Under certain circumstances, tumor cell with EGFR signaling increases MHC II expression in the absence of co‐stimulatory factors, driving the differentiation of CD4^+^ T cells into Tregs. Finally, the EGFR signaling of Tregs protects FOXP3 from degradation by inhibiting GSK3β, thereby enhancing the immune‐suppressive function of Tregs. The EGFR signaling of CD4^+^ T cells promotes Glut1 expression via the RAS‐TBK1 pathway, inducing the Warburg effect and promoting CD4^+^ T cell apoptosis.

These findings highlight that different EGFR mutations can create inconsistent immune microenvironments depending on the context. While some studies suggest that EGFR mutations may impair immune responses, others have shown no significant difference in CD8^+^ T cell density between EGFR mutant and wild‐type tumors.^[^ ^316^ ^]^ Overall, EGFR mutations exert multiple effects on TME, affecting immune cell composition and function, and ultimately impacting responses to immunotherapy. Next, we will specifically introduce the effects of EGFR on tumor cells and immune cells, respectively, which is illustrated in **Figure** **5**.

##### EGFR Expression in Tumors Affects Immune Cells—Macrophage

It was reported in multiple kinds of cancers, tumor‐associated macrophage (TAM) is associated with resistance to ICBs,^[^ ^317^ ^]^ and their interaction with EGFR signaling has significant implications for cancer progression and treatment response. Detailed mechanisms are illustrated in Figure 5A.

##### 1) EGFR Mutation and Macrophage Density

Intratumoral macrophage density was low in tumors harboring EGFR mutation.^[^ ^316^, ^318^ ^]^ For instance, in EGFR^mut^ patients, fewer CD68^+^ cells (where CD68 is a pan‐macrophage marker used to identify compartmentalized TAMs^[^ ^319^ ^]^ are observed, along with a lower expression level of VEGFA and matrix metalloproteinase (MMP),^[^ ^320^ ^]^ which emphasizes TAM discriminating the TME between different *EGFR* statuses. In summary, cancer cells promote macrophage infiltration and M2 polarization and express specific molecules to evade macrophage phagocytosis through the EGFR signaling pathway.

##### 2) EGFR‐Mediated Macrophage Polarization

In advanced EGFR^mut^ NSCLC, *EGFR* status affects the polarization of macrophages. The majority of TAMs are of the M2 phenotype (marked by CD163), with rare M1 HLA‐DR‐stained cells.^[^ ^321^ ^]^ EGFR‐activated NSCLC tumors promote ILT4 expression via the EGFR‐AKT and ERK1/2 signaling. ILT4, in turn, promotes macrophage infiltration and M2 polarization.^[^ ^245^ ^]^ Moreover, in NSCLC patients with osimertinib resistance, the EGFR^L792F^ mutation following the acquisition of T790M promotes STAT3 Tyr705 phosphorylation. This leads to an increase in IL‐4 expression and secretion, further promoting macrophage M2 polarization and triggering immune escape.^[^ ^322^ ^]^ Interestingly, epiregulin (EREG) secreted by macrophages promotes EGFR‐TKI resistance via EGFR/ErbB2 heterodimer.^[^ ^323^ ^]^ But in the scRNA sequencing data of the early stage of lung adenocarcinoma (LUAD) harboring EGFR mutations, TAMs are not polarized to a distinct state of either M1 or M2.^[^ ^324^ ^]^

##### 3) Macrophage Attraction and Recruitment by EGFR‐Related Signaling

Apart from polarized macrophage, the attraction and recruitment of macrophage also accounts for the ICB response in different *EGFR* status. In glioblastoma, EGFR and EGFRvIII cooperate to induce macrophage attraction via upregulating CCL2, with KRAS serving as a critical signaling intermediate.^[^ ^325^ ^]^ CCL2 expression is also increased in EGFR‐ and/or HER2‐positive breast cancer, leading to increased TAM recruitment and motility.^[^ ^326^ ^]^ CCL2 is a target gene downstream of pleckstrin‐2 (PLEK2) /EGFR signaling, promoting TAM infiltration and tumor cell growth. PLEK2 could interact with the kinase domain of EGFR and suppress EGFR ubiquitination mediated by c‐CBL, leading to constitutive activation of EGFR signaling.^[^ ^327^ ^]^ GBM with higher EGFR levels displayed higher expression of vascular cell adhesion molecule‐1 (VCAM‐1), mediated by the p38/STAT pathway^[^ ^328^ ^]^ or through protein kinase (PKC) and NF‐κB,^[^ ^329^ ^]^ thereby promoting macrophage infiltration and glioblastoma invasion.

##### 4) Evasion of Macrophage Phagocytosis by EGFR Mutant Cancer Cells

EGFR mutant NSCLC tumors evade macrophage phagocytosis via activating the AKT/NF‐κB and ERK/c‐Myc signaling pathways to increase CD47 expression,^[^ ^251^ ^]^ which acts as a signal of “don't eat me,” preventing macrophages from phagocytosing cancer cells.^[^ ^247^ ^]^

##### EGFR Expression in Tumors Affects Immune Cells—CD8^+^ T Cell

Cytotoxic effector CD8^+^ T cells are a key component of the adaptive immune system and play an important role in immune surveillance and defense against infections and cancer.^[^ ^330^ ^]^

Overall, tumors with *EGFR* mutations often display lower infiltration and disorder of CD8^+^ T cell,^[^ ^216^, ^293^, ^331^ ^]^ which mainly explained as following three aspects (Figure 5B): 1) EGFR activation affects recognition of CD8^+^ T cell via reducing the expression of antigen‐presenting molecules such as neoantigens and self MHC,^[^ ^208^, ^332^ ^]^ thereby decreasing the likelihood of recognition by T cells; 2) EGFR activation can reduce the CD8^+^ T cell infiltration into tumors via modulating expression level of cytokines and chemokines. For example, EGFR activation downregulates CXCL10 via inhibiting IRF1 expression through the downstream signal PI3K/AKT,^[^ ^230^, ^333^, ^334^ ^]^ and CXCL10 is known to recruit effector CD8^+^ T cells^[^ ^335^, ^336^ ^]^; 3) EGFR activation can stimulate exhaustion of CD8^+^ T cells via expressing immunosuppressive‐related molecules. For example, EGFR^mut^ NSCLC markedly increased IL6 secretion, which is associated with an immunosuppressed phenotype and worse outcome clinically. Inhibiting IL6 can enhance antitumor immunity and sensitize EGFR^mut^ tumors to anti‐PD‐1 therapeutic efficacy via increasing tumor‐infiltrating IFNγ^+^ CD8^+^ T cells.^[^ ^283^ ^]^ And tumor cells express lncRNA (LINC00973) through EGFR/Wnt signaling, which increases CD55/CD59 expression and inhibits the secretion of cytokines required for CD8^+^ T cell activation. In human lung cancer samples, a negative correlation was observed between CD55/59 and CD8^+^ T cell infiltration, and anti‐CD55/CD59 and anti‐PD‐1 showed synergistic tumor‐inhibiting effects.^[^ ^337^ ^]^ EGFR‐activating mutations can also increase the expression of B7‐H4 (another novel immune checkpoint) through the MEK/ERK pathway and downregulate PD‐L1 via PI3K/Akt. Silencing B7‐H4 can reverse the inhibition of CD8^+^ T‐cell function and block the tumor immune escape.^[^ ^338^ ^]^ In summary, tumor cells reduce their chances of being recognized and killed by CD8^+^ T cells and decrease the infiltration and cytotoxicity of CD8^+^ T cells through the EGFR signaling pathway, thereby weakening the effectiveness of immunotherapy.

##### EGFR Expression in Tumors Affects Immune Cells—CD4^+^ T Cells

Tregs are immunosuppressive CD4^+^ T cells that express the major transcription factor Foxp3 and play an important role in maintaining self‐tolerance,^[^ ^339^ ^]^ which contributes to tumor progression and failure of antitumor immunity.^[^ ^340^ ^]^ Compared with EGFR^wt^, LUAD patients with EGFR mutations have higher levels of CD4 and Foxp3.^[^ ^341^ ^]^ Tregs are highly infiltrated in EGFR^mut^ tumors,^[^ ^212^, ^342^ ^]^ the potential mechanism is that EGFR in tumor cells activates cJun/cJun N‐terminal kinase, increasing the expression of CCL22 and recruiting CD4^+^ regulatory T cells.^[^ ^230^ ^]^ And Mucin1 interacts with EGFR to induce Foxp3^+^ Treg cell infiltration via the EGFR/PI3K/Akt signaling pathway.^[^ ^343^ ^]^ EGFR^mut^ tumors expressed substantial levels of MHC II, which recruit CD4^+^ Tregs via CCL17/CCL22/CCR4 axis, forming a Treg‐enriched TME.^[^ ^212^ ^]^ EGFR expressed in HNSCC inhibits the expression of MHC II, while EGFR inhibitors increase the expression of HLA‐DR, while peptide EGFR_875‐889_ peptide was able to induce effective antitumor CD4^+^ T‐cell responses against tumors expressing EGFR.^[^ ^344^ ^]^ Besides, EGFR promotes CD4^+^ T cell activation and apoptosis through the TBK1/Exo84/RalA/Glut1 pathway, which induces the Warburg effect causing immune cell exhaustion eventually.^[^ ^345^ ^]^ Compared with wild‐type tumors, squamous cancers with genetically depletion of EGFR or treated with gefitinib showed fewer infiltrating Foxp3 Treg cells, lower Foxp3 RNA and a lower percentage of CD4 PD1 positive cells.^[^ ^342^ ^]^ Amphiregulin (AREG), a known ligand of EGFR with increased expression in EGFR^mut^ tumors, is critical for Tregs function in vivo.^[^ ^346^, ^347^ ^]^ Using scRNA seq analysis of a transplanted mouse model of engineered B16 melanoma cell line, it was found that AREG‐EGFR axis mediated the crosstalk between IL‐1 receptor like 1 (IL1RL1) ^+^ Treg cells and cancer‐associated fibroblasts (CAFs), which enables Tregs to promote the pro‐fibrotic and immunosuppressive functional status of CAFs.^[^ ^348^ ^]^ The above studies demonstrate that EGFR activation in tumor cells can promote CD4^+^ Tregs infiltration into tumor cells, thereby forming an immunosuppressive microenvironment (Figure 5C).

##### EGFR Expression in Tumors Affects Immune Cells—NK Cell

NK cells are the first line of defense of the human body against cancer cells and viral infections, capable of nonspecific direct killing of tumor cells without the need for antigen sensitization, antibody involvement, or MHC restriction.^[^ ^349^ ^]^ The interaction between EGFR‐related processes and NK cells has significant implications for cancer immunology.

##### 1) EGFR Mutation and NK Infiltration

In EGFR^mut^ tumors, it has fewer infiltrated CD56^dim^ NK cells, which constitute the majority of peripheral blood NK cells.^[^ ^350^, ^351^ ^]^ This reduction in infiltration may impact the overall antitumor immune response, as CD56^dim^ NK cells are crucial for efficient cytotoxicity against tumor cells.

##### 2) EGFR‐TKI Treatment and NK‐Cell‐Mediated Cytotoxicity

Ovarian cancer cells pretreated with anti‐EGFR TKIs exhibit increased sensitivity toward NK cell‐mediated ADCC,^[^ ^352^ ^]^ suggesting EGFR‐TKI treatment can modulate the tumor cell surface properties, making them more susceptible to NK‐cell‐mediated killing. However, in EGFR^mut^ NSCLC with acquired resistance to EGFR‐TKIs, there is an increase in IL‐6 secretion. This elevation in IL‐6 is associated with a decrease in activated NK cells within the tumor. Blockade of IL‐6 enhances the expression of the NK activation marker natural killer group 2 member D (NKG2D) without affecting NK cell proliferation.^[^ ^283^ ^]^

##### 3) EGFR Signaling and NK Cell Activation Receptors

NKG2D is an activating receptor for NK cells, and its ligand UL16 binding protein 1 (ULBP1), plays a crucial role in triggering NK‐cell‐mediated killing. EGFR inhibitors have been reported to enhance the susceptibility of lung cancer cells to NK‐cell‐mediated lysis. This occurs through the induction of ULBP1 by inhibiting the PKC pathway. Conversely, cancer cells may suppress NK cell activation by reducing ULBP1 expression via the EGFR signaling pathway, thereby triggering immune escape.^[^ ^353^ ^]^ Additionally, a novel peptide therapeutic agent, cSNX1.3 is designed to inhibit the reverse transport and nuclear localization of EGFR. In breast cancer cells, nuclear EGFR inhibits NK cell recruitment and cytotoxicity, while treatment with cSNX1.3 significantly improves NK cell recruitment and cytotoxicity, although this newly discovered mechanism requires further in‐depth exploration.^[^ ^354^ ^]^

In summary, cancer cells with activated and mutant EGFR may inhibit NK cell infiltration and cytotoxicity by expressing certain cytokines, and reduce the expression of ligands for NK cell activation receptors in cancer cells, contributing to tumor‐mediated immune escape.

##### EGFR Expression in Tumors Affects Immune Cells—Dendritic Cells

DCs play an essential role as antigen‐presenting cells that influence T cell activation and differentiation. Mature DCs are responsible for initiating immune responses, while immature DCs promote immune tolerance,^[^ ^355^ ^]^ which is regulated by some cytokines, such as granulocyte–macrophage colony‐stimulating factor (GM‐CSF).^[^ ^356^, ^357^ ^]^

It is reported that EGFR^mut^ patients had a higher density of matured DCs,^[^ ^316^, ^350^ ^]^ but the functions of DCs were dampened in EGFR^mut^ TME. For instance, in primary lung cancer with EGFR mutations, plasmacytoid dendritic cells (pDCs) exhibited dysfunction and pro‐tumorigenic features.^[^ ^358^ ^]^ EGFR^mut^ cancer cells may affect DCs by secreting certain factors. Lewis lung cancer (LLC) cells with EGFR E746‐A750 deletion secrete exosomes, inducing anergic DCs to suppress antitumor immunity. A combination of EGFR‐TKI gefitinib and GM‐CSF could recover T cell infiltration and enhance the efficacy of ICBs.^[^ ^359^ ^]^ A similar phenotype was found in the HNSCC cells treated with nimotuzumab (an EGFR monoclonal antibody), which promotes DC maturation,^[^ ^360^ ^]^ suggesting EGFR may inhibit DCs maturation and normal function.

##### EGFR Expression in Immune Cells Affects Immune Cells—Macrophage

EGFR activation in macrophages affects tumor treatment through the following mechanisms. First, EGFR activation in macrophages modulates their own functional state. When cells are infected with bacterial pathogens, EGFR signaling activates the NF‐κB and MAPK1/3 pathways, inducing cytokine production and macrophage activation.^[^ ^361^ ^]^ During sepsis, EGFR phosphorylation is essential for Toll‐like receptor 4 (TLR4)‐mediated macrophage activation.^[^ ^362^ ^]^ Additionally, high‐fat diets upregulate EGFR and its ligand amphibian in adipose tissue macrophages (ATMs), while selective EGFR deletion in ATMs inhibits their proliferation and alleviates obesity.^[^ ^363^ ^]^ Myeloid‐specific EGFR knockout significantly reduces macrophage infiltration and cytokine expression,^[^ ^364^ ^]^ highlighting EGFR's role in macrophage function. Furthermore, lipopolysaccharide‐induced EGFR upregulation on macrophage surfaces is facilitated by Rab10, and inhibiting EGFR promotes M2 polarization via peroxisome proliferator‐activated receptor gamma (PPARγ)‐mediated glutamine metabolism, shifting the macrophage balance from M1 to M2.^[^ ^365^ ^]^ A similar phenotype is observed in pancreatic cancer, where regenerating islet‐derived protein 4 (REG4) secreted by cancer cells activates the EGFR/AKT/cAMP response element‐binding protein (CREB) pathway in macrophages, promoting M2 polarization.^[^ ^366^ ^]^

Second, activated EGFR signaling in macrophages drives the secretion of cytokines to affect tumor cells. For example, heparin‐binding EGF‐like growth factor (HB‐EGF) secreted by the TAMs enhanced tumor cell motility and invasion by activating EGFR, correlating with poor prognosis.^[^ ^367^ ^]^ EGFR activation in macrophages also promotes pro‐inflammatory cytokine production via MAPK1/3 and NF‐κB signaling, while EGFR‐deficient macrophages exhibit impaired Th1 and Th17 adaptive immune responses to bacterial infection.^[^ ^361^ ^]^ In addition, in Kupffer cells (resident liver macrophages), IL6 production is strictly EGFR‐dependent. IL‐1β from liver cancer cells activates EGFR signaling in Kupffer cells via ADAM17 (also known as TACE), leading to IL‐6 production through JNK, p38, and IKK signaling. IL‐6 then binds to IL‐6R on liver cancer cells, promoting their proliferation.^[^ ^368^ ^]^ In colorectal cancer, CD68^+^ /EGFR^+^ macrophages do not affect overall survival, while the number of CD11b^+^ /EGFR^+^ myeloid cells is associated with poor prognosis in patients with metastatic diseases. EGFR activation in myeloid cells increases STAT3 phosphorylation and IL‐6 expression, promoting colorectal cancer cell proliferation^[^ ^369^ ^]^ (Figure 5A).

In summary, EGFR activation in macrophages has a profound impact on macrophage function, including activation, infiltration, and polarization, secretes some cytokines to promote tumor cell proliferation and metastasis, and affects the expression of inflammation‐related genes in other immune cells, which in turn can significantly influence tumor treatment outcomes.

##### EGFR Expression in Immune Cells Affects Immune Cells—CD8^+^ T Cells

Currently, there are no reports of CD8^+^ T cells naturally expressing EGFR, but studies have documented the overexpression of EGFR in CD8^+^ T cells. Lasarte et al. transduced retroviruses into CD8^+^ T cells to induce EGFR expression and subsequently activated the EGFR pathway using EGFR ligands. They found that CD8^+^ T cells overexpressing EGFR exhibited enhanced proliferation compared to wild‐type CD8^+^ T cells, producing higher levels of IFN‐γ and TNF‐α while displaying lower expression levels of exhaustion‐related markers, such as PD1, TIGIT, and LAG3. This significantly enhanced the antitumor capacity of CD8^+^ T cells.^[^ ^370^ ^]^ Therefore, increasing the infiltration of EGFR‐positive CD8^+^ T cells into tumors may contribute to improved immunotherapy efficacy.

##### EGFR Expression in Immune Cells Affects Immune Cells—CD4^+^ T Cells

EGFR is also expressed on CD4^+^ T cells; selective inhibition in CD4^+^ T cells decreases their proliferation, activity, and the production of cytokines like interferon‐γ, IL4, and IL2.^[^ ^371^ ^]^ This indicates that the normal function of EGFR in CD4^+^ T cells is crucial for maintaining their immunological activities. In the study of EGFR affecting CD4^+^ T cells, there are more studies on its impact on Tregs. Xia et al. found that AREG secreted by cancer cells could bind to Tregs and activate EGFR signaling, which regulates posttranslational modification of Foxp3 protein through glycogen synthase kinase‐3 beta (GSK‐3β), enhancing the immunosuppressive function of Treg cells.^[^ ^372^ ^]^ And AREG/EGFR axis has also been identified as a potential therapeutic target in lupus nephritis, where it downregulates pathogenic CD4^+^ T helper cell responses.^[^ ^373^ ^]^ Compared to EGFR‐negative Tregs, EGFR‐positive Tregs exhibit greater immunosuppressive capabilities. They display higher expression of immunosuppressive molecules and more robust inhibition of CD8^+^ T cells when stimulated by AREG.^[^ ^374^ ^]^ This suggests that EGFR activation in Tregs plays a significant role in promoting an immunosuppressive tumor microenvironment.

In the context of inflammation, EGFR on CD4^+^ T cells has been shown to have a regulatory role. For example, in mice with influenza virus infection, there is an increase in EGFR expression on the lung‐infiltrating CD4^+^ T cells, in which IL17 binds to recruited EGFR in the receptor complex and activates the scaffold protein TNF receptor‐associated factor 4 (TRAF4). TRAF4 activation may limit IL‐17‐mediated pathogenic processes, thereby alleviating inflammation.^[^ ^375^ ^]^ This highlights the potential of EGFR in CD4^+^ T cells as a regulator of inflammation during viral infections.

EGFR signaling in CD4^+^ T cells can also promote their apoptosis. Tang et al. reported that in sepsis, EGFR promotes CD4^+^ T cell apoptosis by facilitating glucose transporter 1 (Glut1) translocation to the cell surface via the TANK‐binding kinase 1 (TBK1)/Exo84/RalA pathway, and EGFR inhibitors effectively alleviated the decrease in total CD4^+^ T cells in septic mice^[^ ^345^ ^]^ (Figure 5C).

##### EGFR Expression in Immune Cells Affects Immune Cells—Others: Eosinophils, Platelets, and Dendritic Cells

IL‐33 binds to its receptors on memory Th2 cells, promoting the secretion of amphiregulin. Amphiregulin then binds to EGFR on the surface of eosinophils, stimulating the production of osteoporosis, a key inflammatory protein that promotes pulmonary fibrosis.^[^ ^376^ ^]^

Tang et al. revealed that platelets enhance the expression of reactive oxygen species (ROS), inducible nitric oxide synthase (iNOS), and CD64 in bone marrow‐derived macrophages (BMDMs) upon lipopolysaccharide treatment. However, treatment with erlotinib on platelets significantly reduced the production of ROS, iNOS, and CD64 in BMDMs. These findings strongly suggest that platelet EGFR plays a positive role in platelet‐mediated M1 macrophage activation.^[^ ^377^ ^]^

The expression of EGFR on DCs can promote the activation of several signaling pathways, including MAPK, KB1/AMPK, PI3K/AKT/mTOR/S6K as well as NF‐κB, in response to ultraviolet (UV) radiation. This activation protects DCs from UV‐induced apoptosis.^[^ ^378^ ^]^

EGFR expression in immune cells profoundly influences their functions and tumor microenvironment dynamics. In macrophages, EGFR activation modulates their polarization (e.g., promoting M2 polarization via PPARγ‐mediated metabolism), cytokine secretion (e.g., HB‐EGF, IL‐6), and infiltration, thereby supporting tumor progression and inflammation. For instance, EGFR signaling in macrophages activates NF‐κB and MAPK pathways to enhance pro‐inflammatory responses or activates AKT/CREB to promote M2 polarization in cancer. In CD8^+^ T cells, while natural EGFR expression is absent, retroviral‐induced EGFR overexpression enhances proliferation and antitumor capacity by reducing exhaustion markers, including PD1 and TIGIT, and boosting IFN‐γ/TNF‐α secretion. In CD4^+^ T cells, EGFR is critical for maintaining proliferation and cytokine production (IFN‐γ, IL‐4, IL‐2). In Tregs, EGFR activation via AREG/GSK‐3β enhances immunosuppressive function by modifying Foxp3. EGFR in CD4^+^ T cells also regulates inflammation (e.g., limiting IL‐17‐mediated pathology) and promotes apoptosis in sepsis via Glut1 translocation. Other cells, like eosinophils, rely on EGFR for amphiregulin‐induced fibrosis; platelets enhance M1 macrophage activation via EGFR, and dendritic cells employ EGFR to activate survival pathways (MAPK, PI3K/AKT) under UV stress. Collectively, EGFR orchestrates diverse immune cell behaviors, impacting tumor immunity, inflammation, and host defense.

## 5 Strategies to Improve the Efficacy of Immunotherapy Against EGFR Overexpression and Mutation

**Figure 6:**
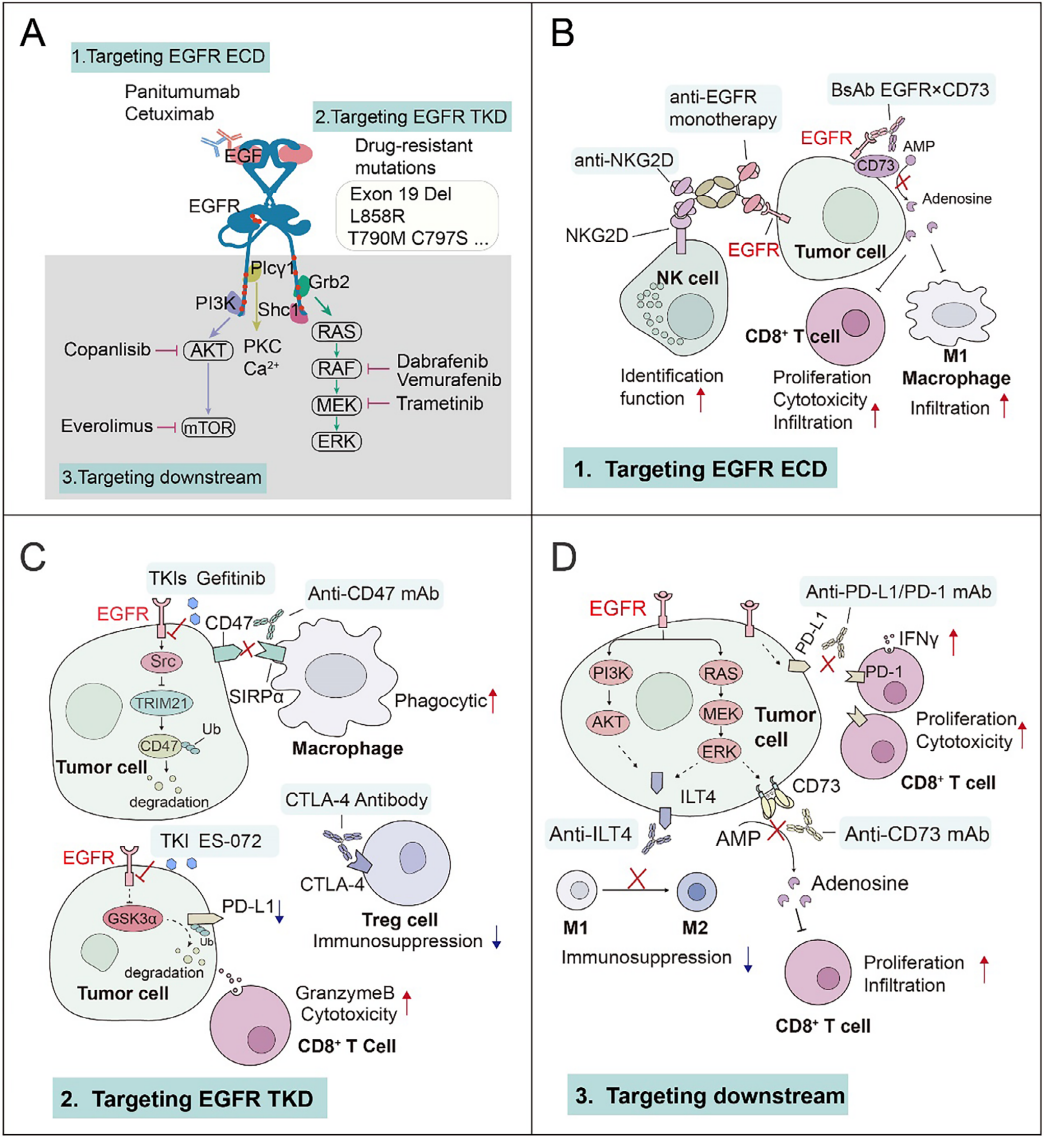
Strategies to improve the efficacy of immunotherapy against EGFR overexpression and mutation. A) Anti‐cancer drugs targeting EGFR signaling. (1) Anti‐EGFR monoclonal antibodies (mAbs) targeting EGFR include cetuximab, panitumumab. These mAbs bind to the extracellular domain (ECD) of EGFR and inhibit the binding of ligands to EGFR. (2) EGFR‐TKIs target the intracellular tyrosine kinase domain to inhibit autophosphorylation of EGFR from the first to fourth generations, mainly focusing on the different drug‐resistant mutations. (3) Targeting downstream of EGFR signaling, mainly including PI3K, mTOR, RAF, and MEK. B) Immunotherapy combined with targeting EGFR at the extracellular domain. Bispecific antibodies target both EGFR and CD73 in tumor cells, inhibiting the EGFR signaling pathway and blocking the conversion of AMP to adenosine. Adenosine is known to inhibit M1 macrophage infiltration and CD8^+^ T cell proliferation, cytotoxicity, and infiltration. Antibodies that target NKG2D on NK cells and EGFR on tumor cells not only promote the activation of NK cells and recognition of EGFR‐overexpressing tumor cells, but also inhibit the EGFR signaling of tumor cells. C) Immunotherapy combined with targeting EGFR at the intracellular domain. EGFR TKI gefitinib inhibits the EGFR signaling in tumor cells, which leads to src phosphorylation, in turn inhibiting the E3 ubiquitin ligase TRIM21, promoting CD47 ubiquitination and degradation. Concurrently, anti‐CD47 mAb is employed to block the binding of CD47 in tumor cells to SIRPα in macrophages, further augmenting macrophage phagocytosis. EGFR TKI ES‐072 alleviates the inhibition of GSK3α by suppressing EGFR signaling, ultimately promoting the ubiquitination‐mediated degradation of PD‐L1, and reducing the PD‐L1 level in tumor cells. Simultaneously, CTLA‐4 antibody is used to inhibit the immune‐suppressive function of Tregs. The combined treatment greatly promotes the release of effector molecules such as granzyme B from CD8^+^ T cells, enhancing the cytotoxicity of CD8^+^ T cells against tumor cells. D. Immunotherapy combined with targeting downstream targets of EGFR. EGFR upregulates ILT4 expression in tumor cells through the PI3K‐AKT and ERK pathways. Antibody‐mediated inhibition of ILT4 halts the polarization of M1 macrophages into M2 macrophages, thereby reducing the immunosuppressive function of macrophages. Concurrently, antibody‐based blockade of PD‐L1 and CD8^+^ T‐cell interaction increases the cytotoxicity and proliferation of CD8^+^ T cells. EGFR promotes CD73 expression in tumor cells through the RAS‐MEK‐ERK pathway, while anti‐CD73 mAb is used to impede the conversion of AMP to adenosine (Ado), thereby alleviating Ado‐mediated inhibition on CD8^+^ T cells and promoting CD8^+^ T cell proliferation and infiltration. Anti‐PD‐L1 mAb can block the binding between PD‐L1 in tumor cells and PD‐1 in T cells, thereby increasing the release of effector molecules such as IFNγ and enhancing the cytotoxicity of CD8^+^ T cells.

Immunotherapy has extended treatment options for many patients, yet a significant number still fail to derive benefit from such treatments. The above summarized research indicates that a considerable portion of these non‐responders exhibit EGFR overexpression and mutations, which could be a pivotal factor contributing to unfavorable prognosis. Consequently, the effectiveness of immunotherapy alone for this group of patients is limited, and there is a clear necessity to also target EGFR. Here are several strategies that have emerged from experimental and clinical studies aimed at enhancing the efficacy of immunotherapy by addressing EGFR overexpression and mutation. Considering the immunosuppressive TME of EGFR^mut^ tumors and its negative effect on response to ICBs, some immune‐modulating strategies to activate TME in combination with ICBs can be further explored. **Table** **4** presents clinical trials of immunotherapy combined with targeted EGFR therapy. Three potential strategies targeting EGFR signaling are illustrated in **Figure** **6**A.

**Table 4:** Clinical outcome of ICBs in cancer patients harboring EGFR^wt^ and EGFR^mut^.

Immunotherapy	EGFR targeted agents	Cancer type	Clinical trial status	Clinical trial number	Ref
Nivolumab (anti‐PD‐1 antibody)	Cetuximab	R/M HNSCC	II	NCT03370276	[379]
Pembrolizumab (anti‐PD‐1 antibody)	Cetuximab	HNSCC	II	NCT03082534	[380]
Pembrolizumab (anti‐PD‐1 antibody)	Cetuximab	Colorectal cancer	Ib/ II	NCT02713373	[385]
Camrelizumab(anti‐PD‐1 antibody)and liposomal irinotecan	Cetuximab	Colorectal cancer	II	ChiCTR1900027573	[387]
Tislelizumab (anti‐PD‐1 antibody) and irinotecan (topoisomerase I inhibitor, chemotherapy)	Cetuximab	Colorectal cancer	II	NCT05278351	[386]
Durvalumab (anti‐PD‐L1 antibody)	Cetuximab	HNSCC	II	NCT03691714	[381]
Avelumab (anti‐PD‐L1 antibody)	Cetuximab	Colorectal cancer	II	NCT04561336	[388]
Ipilimumab (anti‐CTLA‐4 antibody)and radiation therapy	Cetuximab	HNSCC	I	NCT01935921, NCT02777385	[382]
Urelumab (anti‐ CD137 agonist monoclonal antibody)	Cetuximab	Colorectal cancer and HNSCC	Ib	NCT02110082	[465]
CART‐EGFR‐IL13Rα2		Glioblastoma	I	NCT05168423	[400]
Anti‐CD3 x anti‐EGFR bispecific antibody		Pancreatic cancer	I / II	NCT01420874 and NCT02620865	[397]
Nivolumab (anti‐PD‐1 antibody)	Erlotinib	NSCLC	I	NCT01454102	[194]
Pembrolizumab (anti‐PD‐1 antibody)	Afatinib	NSCLC	II	NCT03695510	[411]
Pembrolizumab (anti‐PD‐1 antibody)	Afatinib	Squamous cell carcinoma of the lung	II	NCT03157089	[413]
Atezolizumab (anti‐PD‐L1 antibody)	Erlotinib	NSCLC	Ib	NCT02013219	[201, 461]
Durvalumab (anti‐PD‐L1 antibody)	Osimertinib	NSCLC	Ib	NCT02143466	[199, 415]
Durvalumab (anti‐PD‐L1 antibody)	Osimertinib	NSCLC	III	NCT02454933	[462]
Selumetinib (MEK1/2 inhibitor) or savolitinib (MET‐TKI) or durvalumab	Osimertinib	NSCLC	Ib	NCT02143466	[199]
Durvalumab (anti‐PD‐L1 antibody)	Gefitinib	NSCLC	I	NCT02088112	[198]
Tremelimumab (anti‐CTLA‐4 antibody)	Gefitinib	NSCLC	I	NCT02040064	[417]
HF10 (oncolytic virus) and gemcitabine (Chemotherapy drugs that inhibit DNA synthesis)	Erlotinib	Pancreatic cancer	I	UMIN000010150	[421]

### 5.1 Immunotherapy Combined with Targeting EGFR at the Extracellular Domain

#### 5.1.1 PD‐1/PD‐L1 Monoclonal Antibody Combined with EGFR Monoclonal Antibody

In the context of HNSCC, cetuximab, an EGFR monoclonal antibody, has been approved by the FDA. Multiple studies^[^ ^379^, ^380^, ^381^ ^]^ have explored its combination with ICBs, including anti‐PD‐(L)1 and anti‐CTLA4 inhibitors. In a study of two cohorts of recurrent and/or metastatic (R/M) HNSCC patients (*n* = 88, NCT03370276),^[^ ^379^ ^]^ the combination of cetuximab and nivolumab was evaluated. The 1‐year OS rates in the two cohorts, which differed in prior systemic therapy exposure, were 50% and 66%, respectively, and the safety profile was acceptable. Notably, the combination therapy was more effective in p16‐negative patients, potentially due to alterations in the TME.

A phase II clinical trial of 33 HNSCC patients, the combination of pembrolizumab and cetuximab had antitumor effects, with an ORR of 45% (95% CI, 28%–62%), and oral mucositis was the most common adverse effect; no deaths occurred.^[^ ^380^ ^]^ And 33 patients with R/M HNSCC patients treated with durvalumab and cetuximab in a phase II clinical trial, an ORR is 39% with tolerable safety, and the treatment was effective even in patients with prior chemotherapy or immunotherapy. In a phase I clinical trial, 18 HNSCC patients first received cetuximab and radiotherapy, followed by ipilimumab, achieving a 3‐year PFS of 61%, 3‐year disease‐free survival (DFS) of 72%, and 3‐year OS of 72%.^[^ ^382^ ^]^ Additionally, in a phase II study of 88 HNSCC patients treated with cetuximab and nivolumab, it was found that the median OS of previously treated R/M HNSCC patients was 11.4 months, while that of previously untreated patients was 20.2 months, highlighting the antitumor effects and the advantage of early combined‐antibody use.^[^ ^379^ ^]^ However, it pointed out the limitations of such studies, where the ORR and survival data often did not reach statistically significant improvements, and the efficacy of combination therapy might overlap with monotherapy, with treatment sequence also influencing prognosis.^[^ ^383^ ^]^

Beyond HNSCC, other cancer types have also been explored. Cutaneous squamous cell carcinoma (cSCC) patients who received a combination therapy of weekly cetuximab and bi‐weekly nivolumab showed remarkable tumor regression over time and were completely relieved at six months. Positron emission tomography/computed tomography (PET/CT) scans showed that tumor activity had disappeared at eight months, and there was still no recurrence after one year, though large‐scale validation is needed.^[^ ^384^ ^]^ For colorectal cancer, in a phase 1b/II clinical study of 45 refractory mCRC patients with wild‐type RAS, an increase in intratumoral CTLs was observed with the cetuximab–pembrolizumab combination, despite no significant ORR or PFS improvement.^[^ ^385^ ^]^ The combination of cetuximab, tislelizumab, and irinotecan (topoisomerase I inhibitor) was tested in a phase II clinical trial in patients with RAS wild‐type mCRC, which showed longer overall survival and better clinical efficacy.^[^ ^386^ ^]^ Similarly, it has been reported that a median PFS of 6.9 months (95% CI: 2.6–11.2) and a median OS of 15.1 months (95% CI: 6.1–24.0) in 16 such patients treated with cetuximab, camrelizumab and irinotecan liposomes every 2 weeks until disease progression, intolerable toxicity, or patient refusal of treatment occurred.^[^ ^387^ ^]^ In a phase II single‐arm trial, 77 chemotherapy‐resistant RAS wild‐type mCRC patients treated with a combination of avelumab and cetuximab had a median OS of 11.6 months and a median PFS of 3.6 months.^[^ ^388^ ^]^

In NSCLC, an ORR of 23.4% in 64 patients treated with necromumab and pembrolizumab was reported, demonstrating a certain therapeutic effect and testing the maximum tolerated dose.^[^ ^389^ ^]^ In a nasopharyngeal carcinoma case,^[^ ^390^ ^]^ a 55‐year‐old patient intolerant to chemotherapy achieved complete remission after concurrent radiotherapy with the combination of anti‐PD‐1 antibody and cetuximab. The patient's symptoms improved, the tumor shrank, and complete remission was achieved 2 months after the end of radiotherapy, which can be further promoted to more cancer treatments.

Collectively, these studies suggest that PD‐1/PD‐L1 and EGFR antibody combinations can enhance antitumor immunity, particularly in HNSCC and cSCC, but efficacy depends on tumor microenvironment (e.g., p16 status), treatment history, and inclusion of adjunct therapies like chemotherapy or radiotherapy. While early‐phase trials demonstrate feasibility and biologic rationale, larger randomized studies are needed to validate survival benefits, optimize sequencing, and identify predictive biomarkers.

#### 5.1.2 Bispecific Antibody

Lu et al. reported a bispecific antibody, IgTT‐1E, which combines a dual‐targeting tandem trimer with the human IgG1 hinge and Fc region to target both PD‐L1 and EGFR. This approach not only alleviated the reduction of T cell proliferation and cytotoxic activity induced by the binding of tumor cell PD‐L1 to T cell PD‐1, but also inhibited the excessive proliferation of tumor cells triggered by the activation of the EGFR pathway via ligands such as EGF and amphiregulin. Additionally, it also enhanced the antigen‐specific antibody‐dependent phagocytosis of macrophages against tumor cells, associated with a significantly increased population of CD8^+^ T cells in two humanized mouse models.^[^ ^391^ ^]^

Amivantamab is a bispecific antibody that targets EGFR and mesenchymal‐epithelial transition (MET) receptor,^[^ ^392^ ^]^ which is the first approved targeted therapy for advanced NSCLC patients with an EGFR exon 20 insertion mutation in 2021. This multicohort clinical trial (CHRYSALIS, NCT 02609776) showed that co‐targeting c‐Met and EGFR significantly increased the ORR and durable response, with an ORR of 40% and a median duration of response of 11.1 months.^[^ ^393^ ^]^ In addition to NSCLC, there is another clinical trial focused on the efficiency and safety of Amivantamab in EGFR‐ or MET‐amplified esophagogastric cancer (NCT05117931). Vγ9Vδ2 T cells are effector cells with antitumor efficacy that can recognize target cells in an HLA‐independent manner. An EGFR‐Vδ2 bispecific T‐cell engagers (bsTCE) were developed to activate Vγ9Vδ2 T cells and selectively kill EGFR‐overexpressing tumor cells, which promoted the downstream activation of CD4^+^, CD8^+^ T cells and NK cells, but not Tregs. Compared with conventional T cells from healthy donors, cancer patient Vγ9Vδ2 T cells have a distinct inhibitory immune‐checkpoint receptor expression pattern associated with lower PD‐1, LAG‐3, and TIM‐3, which may be relatively resistant to ICB‐mediated inhibition, improving the efficacy of immunotherapy.^[^ ^394^ ^]^

NKG2D is an activating receptor expressed on the surface of NK cells, which can recognize and bind to the ligand NKG2DL on the surface of cancer cells to activate NK cells. Wels et al. designed NKG2D CAR‐engineered NK cells overexpressing NKG2D and designed two bispecific antibodies: targeting NKG2D and EGFR, and NKG2D and ErbB2, respectively. The combination of the three will greatly enhance the cytotoxicity of NK cells against cancer cells overexpressing EGFR or ErbB2.^[^ ^395^ ^]^

The efficacy of conventional monospecific CD73 antibodies may be limited. Helfrich et al. designed a novel tetravalent bispecific antibody that targets both CD73 and EGFR, which demonstrated a significant ability to reactivate the anticancer potential of cytotoxic T cells suppressed by adenosine and concurrently counteracted cancer cell‐surface CD73‐mediated and EGFR‐mediated pro‐oncogenic activities.^[^ ^396^ ^]^ A clinical study showed that a bispecific antibody targeting CD3 and EGFR can activate CD8^+^ T cells, promote their expression of IFN‐γ, and enhance their cytotoxicity against EGFR‐overexpressing cancer cells, thereby greatly improving the time to progress and overall survival of pancreatic cancer patients.^[^ ^397^ ^]^

#### 5.1.3 Engineering Modification of Immune Cells Combined with EGFR Monoclonal Antibody

Chimeric antigen receptor T cells (CAR‐T cells) are genetically engineered T cells that integrate a single‐chain variable fragment (scFv) recognizing tumor‐associated antigens with TCR signaling and co‐stimulatory domains (e.g., CD28, OX40, CD137) into a single molecule. This enables specific recognition and killing of tumor cells without relying on MHC. While CAR‐T cells have shown success in B‐cell malignancies, their application in solid tumors faces challenges, including fatal toxicity from off‐target recognition (e.g., targeting ERBB2/HER2) and immunosuppressive factors in the TME that limit efficacy.^[^ ^398^ ^]^ The high expression of EGFR in many tumor cells presents a potential target for therapeutic intervention. Lu et al. designed a novel PTG‐T16R‐scFv‐CAR‐T cell targeting EGFR and B7 homolog 3 protein (B7H3), which simultaneously inhibited expression of PD‐1, TIM‐3, Tight, TGF‐β receptor, IL‐10 receptor, and IL‐6 receptor with multiple shRNA clusters, enabling CAR‐T cells to escape most of the interference of tumor cells. This therapy greatly improves the activity of CAR‐T cells and improves the survival rate of cholangiocarcinoma model mice in vivo.^[^ ^399^ ^]^ Another CAR T cell therapy targeting EGFR and interleukin‐13 receptor α2 (IL13Rα2) was applied in recurrent glioblastoma, whose median OS is less than 1 year. The design of scFv antibody targeting the EGFR is epitope 806, which is a hidden and conformational epitope that becomes predominantly accessible under conditions of EGFR dysregulation. Currently, a recent phase I trial (NCT05168423) testing the safety and maximum tolerated dose is ongoing. The sample size treated thus far is small, with six patients, and the follow‐up duration is relatively low, but the overall phenotype is a reduction in the tumor sizes, so confirmation with more patients and longer follow‐up is needed.^[^ ^400^ ^]^ Another study demonstrated that directly incorporating cetuximab into CAR‐T cells using a lentiviral vector can effectively target EGFR‐positive cancer cells with high specificity. This approach provides valuable insights for developing immunotherapies that reduce toxicity and enhance efficacy.^[^ ^401^ ^]^

Compared with NK cells transduced with an empty vector, ready‐to‐use EGFR‐CAR NK cells demonstrated enhanced tumor cell killing capabilities. The combination therapy of OV‐IL15C (an oncolytic virus expressing protein that includes human IL‐15 and IL‐15Rα sushi domain) and EGFR‐CAR NK cells exhibited synergistic effects over individual monotherapies in glioblastoma. This synergy was associated with increased intracranial infiltration and activation of NK and CD8^+^ T cells, as well as enhanced persistence of CAR NK cells within an immunocompetent model.^[^ ^402^ ^]^ Compared to conventional NK cells, memory‐like (ML) NK cells exhibit enhanced production of IFN‐γ and TNF, stronger degranulation and cytotoxicity, and superior in vivo control of HNSCC tumors. ML‐NK cells engineered with anti‐EphA2 CAR show improved antitumor efficacy, as EphA2 is frequently overexpressed in many solid tumors. Additionally, the combination of ML‐NK cells and cetuximab significantly enhances the antitumor response against HNSCC cells, warranting further investigation in clinical trials.^[^ ^403^ ^]^

As an Fc receptor for IgG, CD16 plays a crucial role in ADCC, activating the killing function of immune cells against target cells. A point mutation at position 158 of CD16, delivered via retroviral transduction to chimeric receptor (CR) T cells, can increase the release of antitumor‐related cytokines. Combined with cetuximab, this approach significantly inhibits the growth of HCT116 cells (a colorectal cancer cell line with KRAS mutation) and extends disease‐free survival in mice. This provides a new direction for the immunotherapy of KRAS‐mutated cancers.^[^ ^404^ ^]^

Both EGFR antibodies may have complementary effects. Cetuximab (IgG1) and panitumumab (IgG2) are used to treat EGFR‐positive tumors, but panitumumab cannot effectively trigger the ADCC effect of NK cells like cetuximab, while CD32A can bind to both IgG1 and IgG2. Sconocchia et al. designed CD32A131R‐CAR T cells, which showed stronger killing activity against breast cancer cells overexpressing EGFR when used in combination with cetuximab and panitumumab. This provides a new strategy for cell‐based targeted immunotherapy of solid tumors.^[^ ^405^ ^]^

#### 5.1.4 Oncolytic Virus Combined with EGFR Monoclonal Antibody

Immunotherapy and oncolytic virus therapy are potential treatment methods, and their combined treatment effect warrants further investigation. Yu et al. designed EGFR‐CAR NK cells targeting EGFR and oncolytic herpes simplex virus (oHSV), which can selectively replicate in and ultimately lyse tumor cells. The combination of the two therapies does not interfere with each other. In vitro experiments demonstrated that the combination had a more pronounced cytotoxic effect on breast cancer cell lines than either monotherapy. In a mouse model with intracranial inoculation of breast cancer cells, while single‐modality treatment could suppress tumor growth, the combination therapy group exhibited significantly enhanced tumor inhibition and markedly prolonged mouse survival.^[^ ^406^ ^]^ Kasuya et al. found that the combination of cetuximab and oncolytic herpes simplex virus canerpaturev (C‐REV) exerted a stronger antitumor effect by promoting the distribution of C‐REV in the tumor and inhibiting angiogenesis than either of them alone.^[^ ^407^ ^]^ Given that EGFR is expressed or amplified in many cancers, equipping oncolytic viruses with EGFR‐redirection paramyxavirus glycoprotein H/F complex can enhance the targeting ability of oncolytic viruses. In a human glioblastoma xenograft model, oncolytic cytomegalovirus expressing EGFR‐redirection H/F complex demonstrated enhanced antitumor efficacy.^[^ ^408^ ^]^

To overcome the drawback of oncolytic viruses being easily cleared by the immune system and poor tumor targeting, modifications have been made to them. As a modified oncolytic adenovirus (OAdv), ICOVIR15‐cBiTE can express EGFR‐targeted bispecific T cell engager (cBiTE), and is combined with mesenchymal stem cells (MenSCs) derived from blood. The rationale is that MenSCs can efficiently deliver the OAdv to the tumor site. Once cells are infected with ICOVIR15‐cBiTE, they can secrete functional cBiTE, which binds to EGFR‐positive cells and induces T cell‐mediated cytotoxicity against EGFR‐positive cells. In NSG mouse models bearing A549 subcutaneous tumors, the combination of ICOVIR15‐cBiTE and MenSCs significantly inhibited tumor growth. This study provides a promising new strategy for cancer treatment and holds potential for clinical trial evaluation.^[^ ^409^ ^]^

#### 5.1.5 Other Immunotherapy Combined with EGFR Extracellular Structure Targeted Therapy

D2C7‐IT is a novel immunotoxin targeting both EGFR^wt^ and mutant EGFRvIII proteins in glioblastoma, which can also induce T cell activation to produce a secondary immune response. In subcutaneous and intracranial glioma mouse models, the combination of D2C7‐IT and αCTLA‐4/αPD‐1 therapy showed significant tumor regression and was effective against both dmEGFRvIII‐positive and ‐negative tumors. Moreover, the combination therapy induced a systemic antitumor response, significantly increased the survival rate of mice, and some mice had their tumors cured and developed resistance to tumor recurrence.^[^ ^410^ ^]^

The above section introduces the combined therapeutic effects of various immunotherapies and EGFR‐targeted immunotherapies, which enhance the efficacy of immunotherapy by inhibiting the binding of EGFR to its ligands or targeting EGFR to promote specific recognition of EGFR overexpression and mutant tumors. Examples, including BsAb of anti‐EGFR and anti‐CD73, anti‐EGFR monotherapy, and anti‐NKG2D in NK cells, are depicted in Figure 6B. Although some clinical results show no significant synergistic effect, most clinical and preclinical trials have their merits, providing valuable ideas for further research. The following section will introduce the combined therapeutic effects of various immunotherapies and targeted EGFR kinase regions.

### 5.2 Immunotherapy Combined with Targeting EGFR at the Intracellular Domain

The indirect and significant enhancement of tumor immunity by EGFR‐TKIs has been shown to reverse the immunosuppressive TME, making the combination of targeted and immunotherapy feasible.

#### 5.2.1 PD‐(L)1 Monoclonal Antibody Combined with EGFR‐TKI

EGFR TKIs have been found to promote antigen presentation and immunotherapy effects. A clinical study evaluated the combination of afatinib and pembrolizumab in 29 HNSCC patients, achieving an ORR of 41.4%, indicating significant therapeutic efficacy. Kyoto encyclopedia of genes and genomes (KEGG) and gene ontology (GO) analyses of biopsy tissues from nine patients revealed upregulation of gene sets related to antigen processing and presentation, as well as NK cell‐mediated cytotoxicity, while gene sets associated with cell differentiation, generation, and proliferation were downregulated. These findings suggest that afatinib combined with pembrolizumab may enhance immunotherapy efficacy by promoting antigen presentation and immune response, thereby reshaping the tumor microenvironment.^[^ ^411^ ^]^ In a retrospective study of 51 R/M HNSCC patients (IRB approval number: 201710031RINB), this combination therapy demonstrated a low grade 3/4 toxicity rate of 7.8% and a high ORR of 54.9%.^[^ ^412^ ^]^ In a multicenter, non‐randomized, open‐label Phase II study involving 24 patients with SCC who had progressed during or after first‐line chemotherapy, the combination of pembrolizumab and afatinib demonstrated a low ORR of only 12.5%. The Phase II recommended dose (RP2D) was terminated prematurely due to poor response and high discontinuation rates.^[^ ^413^ ^]^ This highlights that the efficacy of pembrolizumab and afatinib may vary significantly across different cancer types, emphasizing the need for further research to better understand their therapeutic potential in diverse oncologic settings.

Erlotinib combined with atezolizumab (anti‐PD‐L1 antibody) in 28 NSCLC patients also achieved a high ORR of 75% and a disease control rate of 90%, demonstrating robust therapeutic effects. Notably, some patients continued to show improvement after discontinuation of atezolizumab, indicating enhanced immune system function and sustained antitumor effects. The safety profile was deemed acceptable, suggesting promising clinical potential for this combination therapy.^[^ ^201^ ^]^

Erlotinib combined with nivolumab in 21 patients with EGFR‐mutant advanced NSCLC also demonstrated clinical benefit with acceptable safety. The ORR was 15% (3/20) among patients previously treated with TKIs, with one additional patient achieving a 61% maximum reduction in target lesions. Notably, 1 TKI‐naive patient achieved a complete response that has been ongoing for over 5 years. These findings suggest that combination therapy may offer stronger therapeutic effects for treatment‐naïve patients compared to immunotherapy for those resistant to EGFR TKI treatment.^[^ ^414^ ^]^

A critical consideration in combination therapy is the potential for side effects. In the TATTON trial, a promising ORR for EGFR‐TKI‐naïve with T790M mutant patients was found in the treatment of osimertinib and durvalumab, but this combination is unsafe due to a high incidence of grade 3/4 toxicity (39%), showing 22% interstitial lung disease (ILD).^[^ ^199^ ^]^ In detail, two groups were set up: Group A patients received durvalumab monotherapy, while Group B was treated with the combination of osimertinib and durvalumab. Group B was discontinued early due to ILD‐related AEs. Prior to discontinuation, Group B demonstrated an ORR of 82%, significantly higher than that Group A's 43%. However, the severe side effects in Group B highlight the critical need to consider the balance between efficacy and safety in combination therapies.^[^ ^415^ ^]^ And gefitinib combined with durvalumab in the treatment of NSCLC patients resulted in 50.0% of patients experiencing serious AEs and 68.8% encountering grade 3/4 AEs. The toxicity profile of this combination was notably higher than that of monotherapy, and no synergistic effect was observed.^[^ ^198^ ^]^

To investigate the interaction between nivolumab and EGFR‐TKIs in the treatment of NSCLC, particularly its impact on the incidence of ILD, adverse events were analyzed in 20 516 patients with NSCLC. Among 5777 patients treated with EGFR‐TKIs alone, 265 (4.59%) developed ILD. In contrast, among 70 patients treated with the combination of EGFR‐TKIs and nivolumab, 18 (25.7%) experienced ILD. Although the combination therapy demonstrated a potential synergistic advantage, the significantly higher incidence of ILD highlights the increased risk associated with concurrent use. Therefore, careful monitoring for ILD is warranted when combining nivolumab with EGFR‐TKIs.^[^ ^203^ ^]^

Given the challenge of the potential toxicity of combination therapy, the optimal sequence, time interval, and dosage of administration should take into consideration. For example, immune‐related adverse events (irAEs) were observed in those who received PD‐(L)1 inhibitors sequentially with osimertinib. In contrast, no severe irAEs were reported when osimertinib was administered prior to PD‐(L)1 inhibitors (0/29) or when other EGFR‐TKIs (such as afatinib or erlotinib) were used following PD‐(L)1 blockade (0/27).^[^ ^204^ ^]^

This underscores that different drug combinations can yield varied outcomes, even for the same disease, emphasizing the importance of tailored treatment strategies and vigilant monitoring of adverse events.

#### 5.2.2 Other Monoclonal Antibodies Combined with EGFR‐TKIs

Using a high‐throughput drug screen, it was found that EGFR inhibitors such as ES‐072 induce PD‐L1 degradation, suggesting a combination of EGFR inhibitors might boost antitumor immunity and enhance ICB response. ES‐072, an irreversible mutant‐selective EGFR TKI, promotes PD‐L1 ubiquitination and proteasomal degradation following GSK3α‐mediated phosphorylation of Ser279/Ser283. Combination therapy with anti‐CTLA4 and ES‐072 significantly increased the total and activated level of CD8^+^ cytotoxic T cells in the tumor compared with monotherapy, displaying a greater synergistic effect.^[^ ^416^ ^]^ However, in clinical trials, the combination of anti‐CTLA4 antibodies and EGFR‐TKIs has not demonstrated satisfactory therapeutic effects. Planchard et al. conducted a study involving 27 NSCLC patients treated with the combination of gefitinib and tremelimumab (anti‐CTLA4 antibody). All patients experienced AEs, and the best overall response was stable disease (SD) in 72% of patients. Treatment was discontinued in all patients, primarily due to disease progression (63%), highlighting the limited efficacy of this combination.^[^ ^417^ ^]^

Osimertinib, a third‐generation of EGFR TKI, induced higher CD47 expression in EGFR^mut^ NSCLC HCC827 and NCI‐H1975 cells via NF‐κB signaling, and it also reduced PD‐L1 expression in cancer cells. While anti‐CD47 antibody (B6H12) can efficiently block the interaction of CD47 and SIRPα, the combination with osimertinib dramatically increased the phagocytosis in vitro and enhanced the antitumor effect in vivo.^[^ ^248^ ^]^ Similarly, EGFR‐sensitizing mutation (Ex19Del or L858R) or EGFR‐TKI resistance promotes NSCLC tumors' escape from innate immune attack via upregulating CD47. Compared with monotherapy alone, a combination of anti‐CD47 antibody with EGFR TKI augmented the antitumor efficacy via TAMs reprogramming.^[^ ^251^, ^418^ ^]^

#### 5.2.3 Oncolytic Virus Combined with EGFR‐TKIs

Malignant peripheral nerve sheath tumors (MPNSTs) exhibit hyperactive EGFR signaling. In the xenograft model, oHSV treatment demonstrated superior antitumor efficacy compared to erlotinib alone. However, the combination of oHSV and erlotinib significantly enhanced tumor inhibition compared to either agent used alone.^[^ ^419^ ^]^ In pancreatic cancer, it was found that in the subcutaneously implanted tumors, erlotinib enhances the efficacy of oncolytic herpes simplex virus HF10 by promoting persistent virus distribution. The combination of HF10 and erlotinib significantly reduced tumor growth compared to either agent alone. However, in a peritoneally disseminated model, combination therapy did not improve survival.^[^ ^420^ ^]^ In a phase I clinical trial, pancreatic cancer patients initially received erlotinib and gemcitabine, followed by HF10 after achieving tolerance. Among nine patients, the overall effective rate (PR+SD) was 78%, with a median PFS of 6.3 months and a median OS of 15.5 months. Two patients experienced downstaging and achieved complete surgical remission, indicating that the combination of HF10, erlotinib, and gemcitabine was safe and demonstrated a notable antitumor effect.^[^ ^421^ ^]^ Overall, the combination of oncolytic viruses and EGFR‐TKIs in different tumors is promising but warrants further investigation to optimize therapeutic strategies.

#### 5.2.4 Other Immunotherapies Combined with EGFR‐TKIs

CAR‐T cell therapy has shown significant efficacy in treating hematological malignancies, but some patients may experience recurrence, mainly due to insufficient persistence of CAR‐T cells. TKIs not only exert direct antitumor effects but also possess immunomodulatory potential. Lou et al. demonstrated that pretreating CAR‐T cells with afatinib significantly increased the expression of CD62L (also known as L‐selectin), a molecule highly expressed on T cells that promotes immune cell infiltration, and reduced ROS levels. This enhancement positively impacted T cell viability and proliferation. RNA sequencing further revealed that afatinib pretreatment upregulated memory‐related genes while downregulating exhaustion‐related genes in CAR‐T cells, thereby inhibiting CAR‐T cell exhaustion. In a leukemia mouse model, CAR‐T cells pretreated with afatinib exhibit more efficient antitumor cytotoxicity. These findings provide a new reference for improving the treatment of hematological malignancies and solid tumors using the combination of oncolytic viruses and EGFR‐TKIs.^[^ ^422^ ^]^

The combination of GM‐CSF and gefitinib restores the function of DCs and restores CD8^+^ T cell infiltration in EGFR‐Ex19Del tumors. When combined with anti‐PDL1 therapy, GM‐CSF and gefitinib greatly inhibited the growth of EGFR‐Ex19Del‐driven LLC tumors. In contrast, either agent used alone or in a paired combination did not show significant therapeutic effects. Thus, GM‐CSF and gefitinib markedly enhance the efficacy of anti‐PD‐L1 treatment.^[^ ^359^ ^]^

#### 5.2.5 Dual Inhibitors

Chen et al. synthesized a series of small molecules as a potential dual‐acting PD‐L1/EGFR inhibitor EP26 against glioblastoma. Among them, EP26 displayed the highest inhibitory activity, promoting cell death in vitro and inhibiting tumor growth in vivo, associated with increased CD4^+^ and CD8^+^ T cells in TME, which was more effective than gefitinib and PD‐L1 inhibitor P19. These experiments demonstrate that PD‐L1/EGFR dual inhibitors have promising prospects for immunotherapy.^[^ ^423^ ^]^ In the treatment of HNSCC, the combination of the EGFR inhibitor erlotinib with an anti‐TGF‐β antibody and celecoxib (a COX‐2 inhibitor) significantly enhances antitumor T cell responses by inhibiting TGF‐β and prostaglandin E2 signaling.^[^ ^424^ ^]^

Examples of combinate therapy including gefitinib with anti‐CD47 mAb, ES‐072, and anti‐CTLA4 mAb are depicted in Figure 6C.

### 5.3 Immunotherapy Combined with Targeting EGFR Downstream Pathways

The activation of EGFR in tumor cells can modulate the expression of many genes in downstream signaling pathways to inhibit the migration, recognition, and killing of immune cells, thereby facilitating immune evasion by tumor cells and diminishing the efficacy of immunotherapy. In this context, targeting the downstream EGFR signaling pathways in combination with immunotherapy may enhance therapeutic outcomes.

As a response element of the downstream EGFR signaling pathway, ILT4 impairs T cell function by inducing M2‐like polarization of TAMs and inhibiting T cell proliferation and IFN‐γ secretion. In both in vitro and in vivo models (EGFR‐activated C57BL/6 and humanized NSG models), targeting ILT4 (PIR‐B) or inhibiting PD‐L1 enhances antitumor immunity by augmenting T cell effector functions. However, the combination of these two molecules demonstrates the most significant tumor suppression.^[^ ^245^ ^]^

EGFR^mut^ NSCLC tumors express higher levels of CD73 than EGFR^wt^ tumors, and CD73 expression is regulated by the EGFR/Erk signaling pathway in NSCLC.^[^ ^241^ ^]^ Elevated CD73 levels can inhibit T cell proliferation and effector function, leading to resistance to PD‐(L)1 antibody treatment. Katie et al. demonstrated that either anti‐PD‐L1 or anti‐CD73 antibodies alone failed to inhibit tumor growth in a xenograft mouse model of EGFR‐mutated NSCLC. However, the combination of anti‐PD‐L1 and anti‐CD73 antibodies significantly reduced tumor growth compared to the control group.^[^ ^239^ ^]^

EGFR influences numerous pathways and genes that shape the tumor microenvironment and drive tumor development. While combining immunotherapy with targeting these pathways and genes is theoretically feasible, few studies have explored this approach, with most remaining in the preclinical phase. This may be due to the limited impact of certain pathways or genes, compounded by the fact that these pathways or genes are regulated by multiple factors beyond EGFR. Consequently, current research predominantly focuses on combining immunotherapy with targeting EGFR itself or its kinase activity. However, given that EGFR is expressed in multiple tissue cells and affects multiple pathways, and the side effects of its combination therapy are also significant, as seen in several previous examples. Therefore, targeting specific downstream pathways or genes of EGFR in combination with immunotherapy may offer superior efficacy and tolerability, particularly in treating tumors with specific gene abnormalities.

Examples of targeting EGFR downstream signaling, including ILT4 and CD73, are depicted in Figure 6D.

### 5.4 Immunotherapy Combined with Targeting EGFR Ligands

EGF is one of the ligands of EGFR, and its binding to EGFR activates downstream signaling pathways that promote tumor cell proliferation and survival. Targeting EGF can inhibit the growth of cancer cells overexpressing EGFR. CIMAvax‐EGF is a therapeutic cancer vaccine composed of a human recombinant EGF‐binding carrier protein and Montanide ISA51 as an adjuvant. This vaccine stimulates the production of antibodies that bind to EGF, thereby inhibiting the activation of EGFR and reducing tumor growth. In a phase III clinical trial involving 405 patients with stage IIIB/IV NSCLC, patients were randomized to receive CIMAvax‐EGF (vaccine) or best supportive care (control) 4 to 6 weeks after first‐line chemotherapy. The 5‐year survival rate was 14.4% in the vaccine group compared to 7.9% in the control group. Patients with high levels of EGF had a greater survival benefit, with a median 5‐year survival time of 16.62 and 6.22 months in the vaccine and control groups, respectively. The most common side effects were grade 1 or 2, including injection‐site pain, fever, vomiting, and headache, indicating that CIMAvax‐EGF has an acceptable safety profile.^[^ ^425^ ^]^ These findings suggest that targeting EGF through vaccination can effectively inhibit EGFR‐driven tumor growth and improve survival outcomes in patients with advanced NSCLC. However, there is no research on combining immunotherapy with targeting EGFR ligands currently, possibly due to the complexity of the EGFR signaling network. Given the multiple ligands involved, directly targeting EGFR itself or its kinase activity might appear more straightforward. No matter what, future research can consider the potential synergistic effects of combining immunotherapy with targeted ligand inhibition, especially in patient populations with specific genetic alterations or biomarkers that may predict response.

## 6 Summary

Cancer cells with abnormal activation and mutation of EGFR exhibit numerous characteristics that promote their survival and enable them to evade cancer immunotherapy. First, EGFR signaling affects the tumor itself. EGFR reduces its antigenicity through multiple signaling pathways, thereby decreasing the chance of being recognized by immune cells. Simultaneously, it also promotes EMT and enhances its ability to invade and metastasize, further facilitating immune escape. The EGFR signaling of tumor cells can also promote the expression of immune suppressive molecules on the membrane surface and the release of immune suppressive cytokines into the microenvironment, thereby enhancing the function of immune suppressive cells and inhibiting the function of immune active cells, thus creating an immune suppressive microenvironment.

Currently, many studies report that immune cells can also express EGFR, with a focus on macrophages and CD4^+^ T cells. Overall, these studies have demonstrated that EGFR expression in immune cells can augment their intrinsic function. However, due to limited reports, the precise impact of EGFR expression in immune cells on cancer cell development remains to be comprehensively explored.

Overall, cancer cells with EGFR overexpression and mutation have a detrimental impact on cancer immunotherapy. For instance, some clinical trials of anti‐PD‐L1 immunotherapy have revealed that NSCLC patients with EGFR overexpression and mutation exhibit poorer treatment outcomes compared to those with WT EGFR, which is mainly modulated by the TME. EGFR^mut^ tumors had a noninflamed TME with fewer CD8^+^ T cells and more Tregs, representing lower lymphocyte infiltration associated with low efficacy of ICBs.^[^ ^230^ ^]^ However, no consensus has been observed on the importance of PD‐L1 expression^[^ ^224^, ^426^ ^]^ or TMB^[^ ^427^ ^]^ in EGFR^mut^ tumors. Consequently, it is reasonable to postulate that combination therapy of immunotherapy and EGFR‐targeted drugs might yield better therapeutic effects.

Targeting EGFR enhances immunotherapy response primarily by overcoming the immunosuppressive TME. Some EGFR‐TKIs, such as osimertinib and gefitinib, can inhibit M2 polarization of TAMs and deplete Foxp3^+^ Tregs, thereby overcoming the immunosuppressive TME and creating a more favorable environment for immune cells to function, thus enhancing the efficacy of immunotherapy. Moreover, selecting appropriate immunotherapy strategies based on the EGFR mutation status can synergistically enhance the efficacy of ICBs. For instance, patients with rare EGFR mutations (e.g., G719A) have longer PFS when treated with nivolumab. In contrast, patients with the T790M mutation show variable responses to ICBs. NSCLC patients without the T790M mutation who have received prior EGFR‐TKI treatment tend to benefit more from ICBs with better PFS. Cancer patients harboring L858R benefit more from immunotherapy compared with Ex19Del,^[^ ^428^ ^]^ which coincides with the clinical study of ORIENT‐31,^[^ ^429^ ^]^ ATTLAS.^[^ ^430^ ^]^

It has been shown that patients without EGFR‐TKIs benefit more from immunotherapy compared with TKI experienced, since EGFR‐TKIs foster the TME associated with tumor immune escape via the innate immune signaling pathway of type I IFNs^[^ ^431^ ^]^ or upregulate innate immune checkpoint CD24.^[^ ^432^ ^]^

Uncommon mutations such as G719A, L861Q, and so on have been reported to be associated with more frequent smoking history and tobacco‐related mutational signatures, presenting higher TMB,^[^ ^433^ ^]^ but the potential benefit of immunotherapy in these patients remains an open question because of sample defects or are often excluded from clinical trial statistics. Some retrospective studies suggest that tumors with uncommon EGFR mutations may respond better to immunotherapy.^[^ ^314^, ^434^ ^]^

Critical elements causing immune escape in the EGFR^mut^ patients need to be identified; one candidate is matrix metalloproteinases 11 (MMP11). It was reported that compared with patients with EGFR^wt^, the expression of MMP11 was higher in the EGFR^mut^ patients, including Ex19Del, L858R, and T790M, but there was no difference between patients with uncommon mutations and wild‐type. EGFR^mut^ patients with high expression of MMP11 responded poorly to immunotherapy, representing lower immune cell infiltration of CD8^+^ T cells and NK cells.^[^ ^435^ ^]^

**Figure 7:**
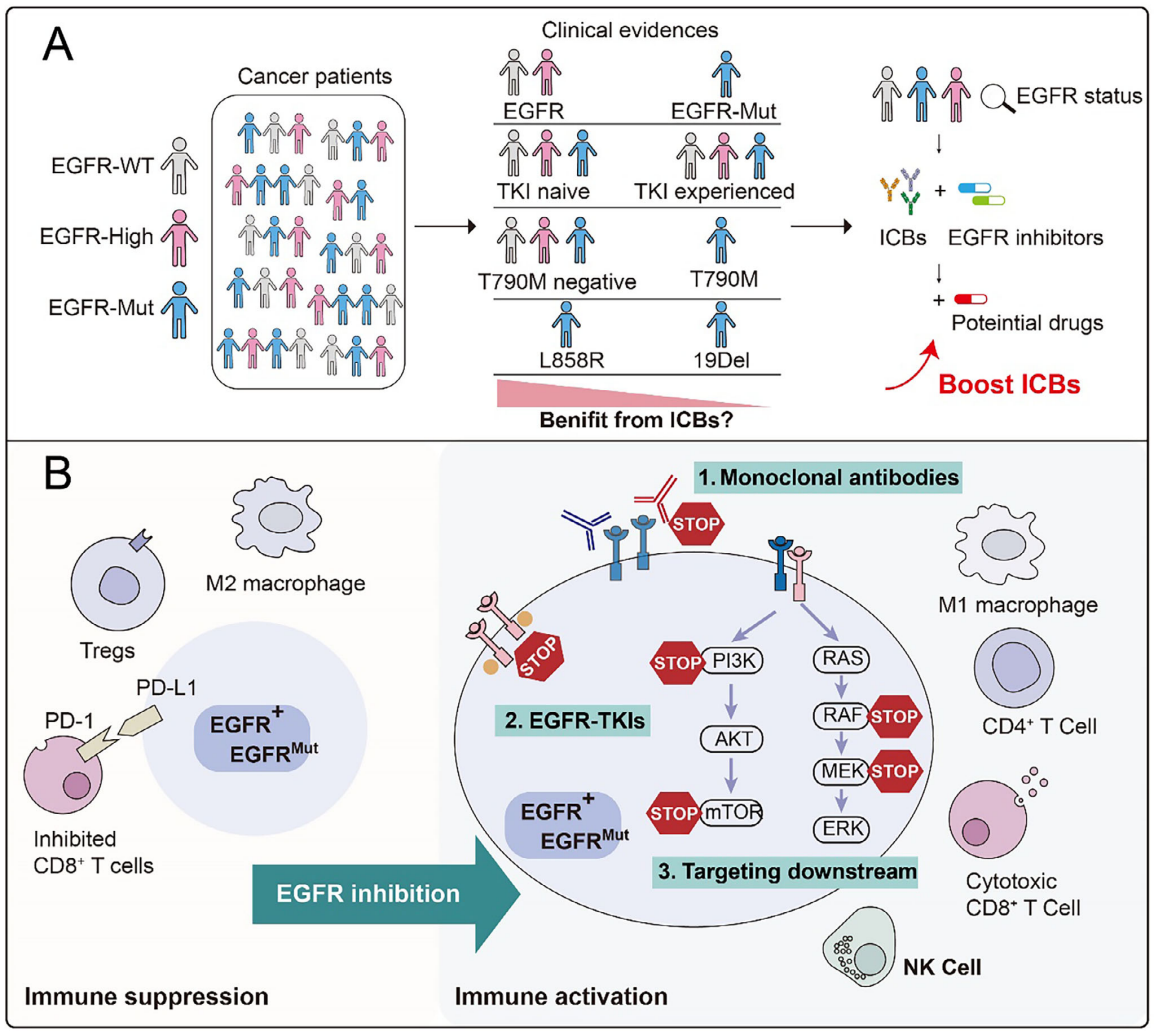
Targeting EGFR reduces immune suppression and remodels the immune tumor microenvironment to boost responses of ICBs. A) Patients with EGFR overexpression and mutations exhibited drug resistance upon EGFR monotherapy and EGFR‐TKIs, and displayed different responses to ICBs. So, how to target EGFR properly to boost the responses of ICBs is the key question. It is worth checking the EGFR status first in the clinical trials, choosing a proper combination with potential drugs, and using the correct medication sequence may help patients benefit from it. B) Given the role of EGFR mutant and overexpression in enhancing immune‐suppressive mechanisms (left panel), the mutant‐specific EGFR inhibition using 3 different strategies results in a reduction of immune‐suppressive cells and an increase in infiltration and activation of the lymphoid population and M1 pro‐inflammatory macrophages (Immune activation; right panel).

Therefore, precise detection of EGFR mutation status and selection of appropriate immunotherapy drugs and timing based on different mutations have the potential to enhance immunotherapy response (**Figure** **7**A).

Current clinical trials of immunotherapy combined with EGFR‐targeted agents primarily concentrate on EGFR itself. This mainly involves the combination of EGFR antibodies and EGFR TKIs, and targeting EGFR downstream signaling. Some of these clinical trials have indeed demonstrated superior therapeutic effects compared to monotherapy. Nevertheless, there are also cases where no synergistic effects were observed. In some extreme cases, several side effects emerged, forcing the termination of the trials. Despite these challenges, combination therapy represents the future direction. Even failed experiments can offer valuable insights for subsequent investigations.

Finally, there is the combination therapy of immunotherapy and targeting downstream targets of EGFR, which currently only exists in the preclinical research stage. There is only one vaccine that targets EGFR ligands, and no immunotherapy has been explored in combination with it. The possible reason for this is that targeting downstream targets or ligands of EGFR is less direct compared to targeting EGFR itself. Given the extensive influence of EGFR signaling, it has manifested significant side effects in certain clinical trials. In contrast, targeting downstream targets or ligands of EGFR may not have a pronounced effect, but the associated side effects could be relatively mild. Moreover, this approach may prove effective in treating cancers with overexpression or mutation of specific downstream targets of EGFR.

Given the role of EGFR mutants and overexpression in enhancing immune‐suppressive mechanisms, various cytokines secreted by the tumor cells promote a TME that is not supportive of immune effector cell function. Following three different strategies to inhibit mutant‐specific EGFR, the tumor immune microenvironment is at least transiently converted to a more active state, providing a window of opportunity where there may be heightened sensitivity to therapies that promote immune responses against the tumor (Figure 7B).

To optimize the combination of EGFR‐targeted agents with immunotherapies, a more comprehensive understanding of how EGFR‐overexpressing and mutant tumor cells reprogram the TME is crucial. Such knowledge holds the potential to significantly enhance clinical outcomes through the strategic use of appropriate combinations of EGFR‐targeted agents and immunotherapies.

## Conflict of Interest

The authors declare no conflict of interest.

## Author Contributions

Y.G. and H.H. contributed equally to this work. Z.Z., H.H. and Y.G. conceived and drafted the manuscript. H.H., Y.G. drew the figures and summarized the tables. S.Q., Y.S., and R.L. participated in the review and editing. Z.Z., L.Z., and F.Z. provided valuable discussion and revised the manuscript. All authors have read and approved the article.
